# Modified Spatially Confined Strategy Enabled Mild Growth Kinetics for Facile Growth Management of Atomically‐Thin Tungsten Disulfides

**DOI:** 10.1002/advs.202205638

**Published:** 2022-11-29

**Authors:** Qun Wang, Shi Wang, Jingyi Li, Yichen Gan, Mengtian Jin, Run Shi, Abbas Amini, Ning Wang, Chun Cheng

**Affiliations:** ^1^ Department of Materials Science and Engineering Southern University of Science and Technology Shenzhen 518055 P. R. China; ^2^ Department of Physics and Center for Quantum Materials Hong Kong University of Science and Technology Hong Kong P. R. China; ^3^ Center for Infrastructure Engineering Western Sydney University Kingswood New South Wales 2751 Australia; ^4^ Guangdong Provincial Key Laboratory of Energy Materials for Electric Power Southern University of Science and Technology Shenzhen 518055 China

**Keywords:** atomically‐thin tungsten disulfide, chemical vapor deposition, growth kinetics, molybdenum/sulfur molar ratio‐time growth diagram, spatially confined growth

## Abstract

Chemical vapor deposition (CVD) has been widely used to produce high quality 2D transitional metal dichalcogenides (2D TMDCs). However, violent evaporation and large diffusivity discrepancy of metal and chalcogen precursors at elevated temperatures often result in poor regulation on X:M molar ratio (M = Mo, W etc.; X = S, Se, and Te), and thus it is rather challenging to achieve the desired products of 2D TMDCs. Here, a modified spatially confined strategy (MSCS) is utilized to suppress the rising S vapor concentration between two aspectant substrates, upon which the lateral/vertical growth of 2D WS_2_ can be selectively regulated via proper S:W zones correspond to greatly broadened time/growth windows. An S:W‐time (SW‐T) growth diagram was thus proposed as a mapping guide for the general understanding of CVD growth of 2D WS_2_ and the design of growth routes for the desired 2D WS_2_. Consequently, a comprehensive growth management of atomically thin WS_2_ is achieved, including the versatile controls of domain size, layer number, and lateral/vertical heterostructures (MoS_2_‐WS_2_). The lateral heterostructures show an enhanced hydrogen evolution reaction performance. This study advances the substantial understanding to the growth kinetics and provides an effective MSCS protocol for growth design and management of 2D TMDCs.

## Graphical abstract

A modified spatially confined strategy create slow rising of S:W to maintain a mild growth kinetics of WS_2_, leading to the facile regulation of selective lateral/vertical growth. Referring to SW‐T diagram, the growth routes for 2D WS_2_ are facilely designed, leading to the versatile growth control of atomically thin WS_2_ on domain size, layer number, and lateral/vertical heterostructures.

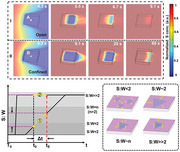

## 1 Introduction

2D transition metal dichalcogenide materials (2D TMDCs) have attracted significant attentions.^[^ ^1^, ^2^, ^3^, ^4^ ^]^ For example, atomically‐thin semiconductive MX_2_ (M = Mo, W etc.; X = S, Se, and Te) with high theoretical carrier mobility (≈1100 cm^−1^ V^−1^ s^−1^)^[^ ^5^ ^]^ and high on/off ratio (10^6^–10^8^),^[^ ^6^, ^7^, ^8^, ^9^ ^]^ have found broad application prospects for the next generation of flexible electronics. The full potentials of these atomically‐thin crystals requires reliable and diversified synthesis of 2D TMDCs, which is essential for the discoveries of their new physics and novel device applications.^[^ ^10^, ^11^, ^12^, ^13^, ^14^ ^]^ Up to now, chemical vapor deposition (CVD) has been widely applied as a mainstream method for the preparation of electronic grade 2D TMDCs, owing to its great potential in low‐cost and scaled‐up productions.^[^ ^1^, ^2^, ^15^ ^]^ For traditional CVD growth of 2D TMDCs, the metal oxide power MO_3_ and chalcogen powder X usually act as reaction sources.^[^ ^7^, ^11^, ^12^ ^]^ In a CVD system, the X:M ratio of vapor has been verified as a crucial reaction kinetics factor that majorly affects the uniform and reproducible growth of 2D TMDCs products.^[^ ^16^, ^17^, ^18^, ^19^ ^]^ However, violent evaporation and large diffusivity discrepancy of metal and chalcogen precursors at elevated temperatures always result in uncontrollable X:M ratio and thus drastic growth kinetics; this phenomenon makes the stable and controllable growth of 2D TMDCs fascinating yet an intractable topic.^[^ ^16^, ^20^ ^]^

Based on the regulation of X:M ratio of vapor, many approaches have been developed to promote the CVD growth of 2D TMDCs with mild reaction kinetics. The first kind of approaches comes from the regulation of M vapor. For example, the physical/chemical barriers, such as inert oxide foams^[^ ^21^ ^]^ and oxide‐inhibitor layers (e.g., SnO_2_, TiO_2_, or Al_2_O_3_),^[^ ^17^ ^]^ and covering metal precursors, were used to suppress the release of M vapor and reduce its concentration gradient on substrates. As a result, appropriate M:X ratio was guaranteed in a large area. However, in these cases, the M vapor concentration gradient was rather at high levels, which degraded the uniformity and domain size of as‐grown products. Alternatively, the metal precursors, such as M foams,^[^ ^22^, ^23^, ^24^ ^]^ M oxides particles or films^[^ ^18^, ^25^, ^26^, ^27^ ^]^ locally supplied a stable and uniform M vapor, and thus improved the growth of 2D TMDCs films.

The second kind of approaches relies on the regulation of X vapor. Chalcogen powder is widely used as X source for the growth of 2D TMDCs films.^[^ ^22^, ^28^, ^29^, ^30^, ^31^, ^32^, ^33^ ^]^ It is found that the products’ morphology, thickness, and domain size were greatly affected by the amount, feeding rate, and exposure time of X.^[^ ^30^, ^34^ ^]^ However, the reproducibility is always a big issue owing to the poor control of the direct evaporation of chalcogen powder. Gaseous X sources, such as H_2_S,^[^ ^18^, ^35^, ^36^, ^37^ ^]^ (C_2_H_5_)_2_S,^[^ ^38^, ^39^ ^]^ etc., are other alternatives but they are highly toxic. Recently, Na_2_SO_4_ was introduced as a novel S precursor, which could be synchronously released with metal precursor during the entire growth stage. But the complicated reactions between Na_2_SO_4_ and WO_3_ led to the limited domain size of 2D products.^[^ ^20^ ^]^ Apart from the selection of appropriate X sources, the space‐confined approach is proposed to greatly suppress the diffusion of S vapor; this setup brings new inspiration to regulate reaction kinetics.^[^ ^40^, ^41^, ^42^ ^]^ Though the above approaches have made impressive progress in the controlled growth of 2D TMDCs, the facile and comprehensive growth design for the desired products of 2D TMDCs is still unavailable because of the lack of the general and deep understanding to the growth kinetics of 2D TMDCs by the classic CVD method, which are greatly influenced by many reaction parameters, such as temperature, pressure, substrate, precursor, etc.^[^ ^15^, ^17^, ^37^ ^]^ As such, the systematical study is crucial, yet absent on the effect of key kinetical factors, such as X:M ratio of vapor, to the growth of 2D TMDCs.

In this work, we developed a modified spatially confined strategy (MSCS) with the combination of local supply of W source to improve the growth management of atomically thin WS_2_ in CVD reaction. By MSCS, the rising S vapor concentration on the surface of space‐confined substrate was greatly suppressed and thus it guaranteed a relatively mild growth kinetics, upon which we proposed a S:W‐time (SW‐T) growth diagram. Referring to this diagram, we designed and implemented the growth routes for comprehensive and flexible modulations on domain size, layer number, and lateral/vertical heterostructures (MoS_2_‐WS_2_) of atomically‐thin WS_2_. The lateral heterostructures showed an enhanced hydrogen evolution reaction (HER) performance. Moreover, the mechanisms of MSCS for regulating the growth kinetics of 2D WS_2_ and heterostructures were discussed in rigorous details.

## 2 Results and Discussion

### 2.1 Simulations of S:W Evolution in Open/Confined Spaces

**Figure 1:**
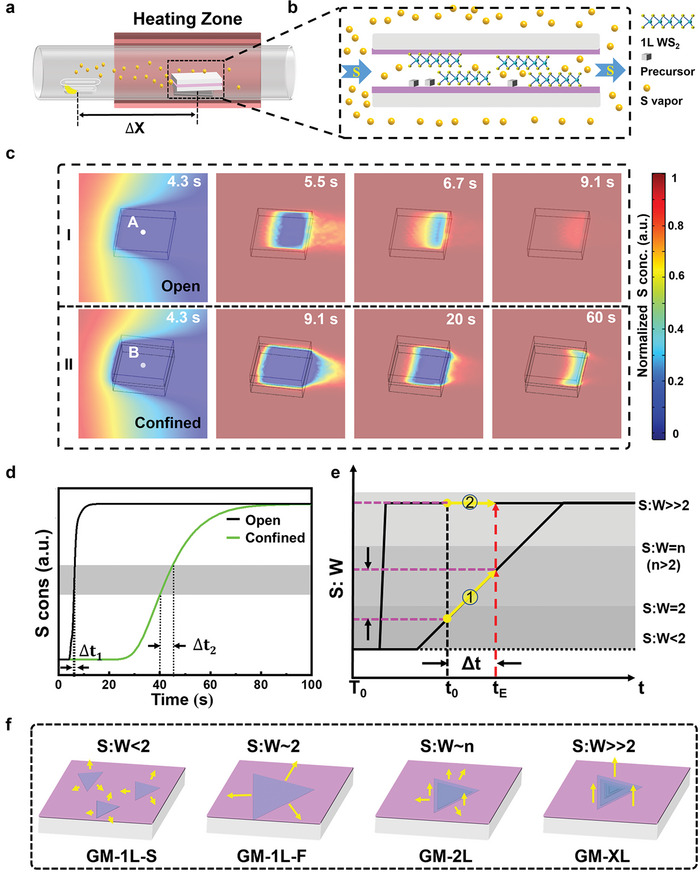
Process of CVD growth: Setup configuration, COMSOL simulation, and SW‐T growth diagram. a) Schematic image of synthesis process of WS_2_ based on the introduced MSCS system. b) Enlarged diagram of the source substrate and the target substrate in the black‐dashed box shown in (a). c) The COMSOL simulation of dynamic distribution of S vapor concentration on the entire substrate based on conventional CVD (CCVD) (I) and MSCS system (II). d) Plots showing the dynamic concentration of S vapor at the center of substrate, derived from the data in (c). Δ*t_1_*, Δ*t_2_* represent the effective time of lateral growth. e) Illustration of the S vapor concentration variation of point A over time. There are four different dynamic periods for S vapor: S:W < 2, S:W ≈ 2, S:W = *n* (*n* > 2), and S:W >> 2. f) Schematic diagram of lateral slow growth (S:W < 2), lateral fast growth (S:W ≈ 2), and vertical growth (S:W >> 2) of WS_2_ in a continuous CVD reaction. Long arrow indicates fast growth speed while short arrow addresses slow growth speed.

**Figure** **1**a,b shows the CVD configuration of MSCS for the growth of 2D WS_2_ (for details refer to Experimental Section). Specially, W precursor (mixture of NaCl and WO_3_, Figure S1a, Supporting Information) was coated on a SiO_2_/Si substrate which was covered by another SiO_2_/Si substrate to create a confined space (Figure S1b, Supporting Information).^[^ ^40^ ^]^ These two aspectant substrates were placed at the center of a high temperature furnace. During the CVD process, the reaction of WO_3_ and NaCl created tungsten compounds (Na*_x_* WO*_y_* and WO*_u_* Cl*_v_*, Section S2, Supporting Information) with low melting points.^[^ ^43^ ^]^ In fact, W vapor was locally supplied and sulfurized to form WS_2_ products on the substrate (Figure S1b, Supporting Information). S:W ratio of vapor is regarded as the most important growth kinetic factor determining the final products on the substrates.^[^ ^16^, ^17^ ^]^ Considering the certain reaction temperature and ambient pressure, the vapor concentration of W on the surface of substrates can be roughly treated as a constant during the growth of WS_2_. Therefore, the products grown on substrates are mainly determined by the distribution of S vapor concentration. For simplicity, plentiful S source is used in the entire reaction (from ≈500 mg for reuse, only 1–2 mg was consumed each time). Two models were built using COMSOL Multi‐Physics software package according to the experimental parameters to qualitatively investigate the distribution evolution of S vapor concentration over time during the reaction (Figure 1c). The uncovered substrate was marked as the “open” mode corresponded to the conventional CVD (CCVD), and the space configuration of two aspectant substrates related to the “confined” mode for MSCS (Figure 1a and Figure S3a, Supporting Information). It was found that S vapor in most region of the substrate in the “confined” mode reached the saturation in 60 s, ≈9 times of “open” mode (6.7 s). This showed a dramatically slow diffusion of S vapor between the two aspectant substrates, therefore, the concentration of S vapor could be well‐suppressed in the confined space for a long time, which resulted in a mild growth kinetics (Movies S1 and S2, Supporting Information).

Points A and B (Figure 1c) are flagged at the center of substrates, to reveal the growth process of 2D WS_2_ for the “open” and “confined” modes. Figure 1d and Figure S3b, Supporting Information, respectively provide the evolution curves of S vapor concentration and gradient in 100 s for points A and B. Noted, the “confined” mode (Point B) shows a much slower rising of S vapor concentration and smaller concentration gradient over time than those of the “open” mode (Point A). Accordingly, the “confined” mode delivers much broader growth windows for any specific S concentration range. For example, Δ*t_2_* is ≈9 times of Δ*t_1_* at the similar S concentration range (marked by gray belt in Figure 1d), where Δ*t* represents the growth window. This endows the “confined” mode with much larger regulation space for the growth of 2D WS_2_ plates compared to the “open” mode. According to the simulated results and a constant concentration of W vapor, a schematic SW‐T growth diagram is proposed as a reference in Figure 1e to describe the design of growth route (GR) along with a timeline for the versatile growth of 2D WS_2_. Based on previous publications, four typical growth modes (GM) at different S:W ratios are proposed in Figure 1f, including GM‐1L‐S (S:W < 2, slow lateral growth of 1L plates), GM‐1L‐F (S:W ≈ 2, fast lateral growth of 1L plates), GM‐2L (S:W ≈ *n*, *n*>2, growth of 2L plates), and GM‐XL (S:W >> 2, vertical growth of XL plates, X > 2).^[^ ^16^ ^]^

For a normal growth on 2D WS_2_, the GR is simply descripted by a yellow line segment in the S:W curve in Figure 1e, where the introduction of W vapor starts at *t*_0_ and ends at *t*_E_ with a time window of Δ*t*. According to the S:W zones and dwelling times that the GR experiences and maintains, the product of as‐grown 2D WS_2_ can be roughly predicted as the integral of growth rate corresponded to S:W ratio along with the GR over the growth time. For example, for similar *t*_0_ and *t*_E_ in Figure 1e, the GRs are distinctly different for the “open” and “confined” modes: GR1 mainly experiences 1L fast growth (GM‐1L‐F) while GR2 carries a multilayer growth (GM‐XL), leading to their corresponding products of small‐sized and multi‐layered plates for CCVD and large‐sized and 1L plates for MSCS. Most importantly, from the SW‐T diagram, GR can be directionally designed by delicately‐adjusted reaction conditions to achieve the versatile modulations of 2D WS_2_ growth with different domain sizes, layer numbers, and heterostructures. In the following, we explain all these desired MSCS products as they deliver a slow growth kinetics and thus endow GRs within the accessible and flexible S:W ranges in the specified timeline.

### 2.2 Comparison of Growth Results in Open and Confined Spaces

**Figure 2:**
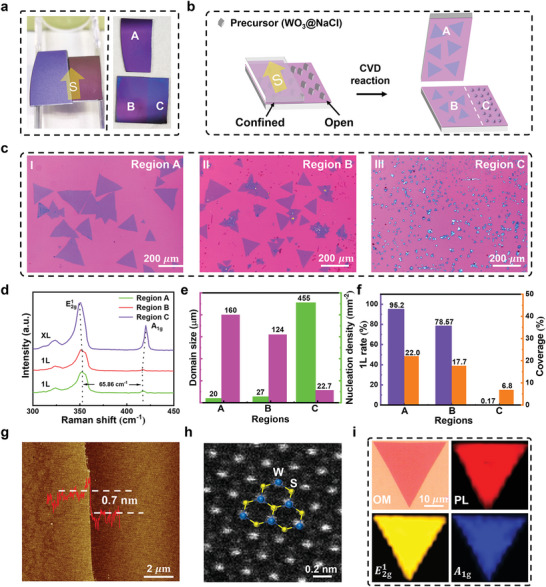
Growth products on open and confined substrates. a) The left inset shows the configuration of MSCS and CCVD modes; The right inset demonstrates the optical images of substrate after sulfurization. b) Schematic diagram of contrast experiment. According to the configuration of two setups of substrates, there are three regions, where regions A and B are “confined” regions, region C is defined as “opened” region. Both yellow arrows in (a) and (b) represent the direction of S vapor during CVD reaction. c) Optical images in (a) for regions A, B, and C. d) Raman spectra captured from substrates A, B, and C. e) Statistical results of average nucleation density and domain size of the regions A, B, and C. f) Statistical results of average 1L rate (number of single layer flakes/total number of flakes) and coverage of regions A, B, and C. g) AFM image of the edge of 1L WS_2_ flake. The inset shows the thickness of the crystal and its corresponding profile. h) HAADF‐STEM image of as‐grown 1L WS_2_ flake. A ball‐stick model of 1L WS_2_ was built for comparison with the STEM image, where indigo balls represent W atoms and yellow balls represent S atoms. i) Optical image of 1L WS_2_ crystal, and the corresponding Raman (351 cm^−1^ for $E_{2g}^{1}$ and 416 cm^−1^ for *A*_1*g*_) and PL mapping (1.97 eV per 633 nm, A exciton).

To verify the effects of MSCS on the regulation of growth kinetics of WS_2_ flakes, we carried out CVD growth experiments on the two aspectant substrates (**Figure** **2**a; for details refer to Experimental Section). The region C is much darker than regions A and B, indicating denser and thicker products. Figure 2b demonstrates the schematic diagram to compare CVD growth of CCVD and MSCS. Figure 2a,b depicts the confined regions A and B corresponded to the MSCS growth while the opened region C is referred to the CCVD growth. In our experiments, NaCl crystals were mixed with WO_3_ at reduced evaporation temperature to act as W precursor.^[^ ^32^ ^]^ The optimized WO_3_/NaCl mass ratio of 2:1 was used in all experiments unless otherwise specified (Section S4, Supporting Information). Figure 2c shows the optical images of typical products of WS_2_ plates of regions A–C, along with their Raman results for layer number identification, and statistic results of domain size, nucleation density, 1L rate, and coverage (Figure 2d–f). As discussed in Section S5, Supporting Information, the open regions A and B mainly contain large, single‐layer WS_2_ flakes with large coverage and 1L rate. On the contrary, the opened region C contains high density growth of WS_2_ flakes with limited domain size (≈20 µm) and uneven thickness (Figure S6a, Supporting Information).

The growth processes of the above obtained products can be deduced based on the observation results in Figure 2c. During the sulfurization, regions A and B underwent a spatially confined growth, where the slowly increasing S precursor concentration on the surface of substrate greatly reduced the nucleation density and extended the effective time of lateral growth of WS_2_ at S:W ≈ 2. On the contrary, region C was directly exposed to ambient atmosphere, where a S:W ratio of >>2 was quickly reached with resultant high‐density nuclei. GS‐XL was performed in region C until W precursor was entirely consumed.^[^ ^44^, ^45^ ^]^ It was found that the growth processes of MSCS (regions A and B) and CCVD (region C), respectively followed GRs 1 and 2 in Figure 1e very well. The distinct growth results were consistent with the predicted ones from the SW‐T growth diagram (Figure 1e), whose validity was primarily verified. The products on the top substrate (Figure 2c(I), region A) were better than those in the bottom substrate (Figure 2c(II), region B). This was due to the slightly large size and cleanness which were resulted from more nucleation sites of the dispersive WO_3_ particles in the bottom substrate besides the spontaneous nucleation. To reduce the influence of WO_3_ particles on the nucleation and growth of WS_2_ flakes, we chose the products of the top substrate for the following study, unless otherwise stated.

The quality of as‐grown WS_2_ plates is comprehensively investigated. The photoluminescence (PL) spectrum (Figure S5b, Supporting Information) in region A shows a strong peak at 1.97 eV (A excitation), as reported before, which confirms the direct band gap of the as‐grown 1L WS_2_ (Figure 2c(I,II)).^[^ ^32^, ^33^, ^34^, ^46^ ^]^ Furthermore, the full width at half maxima (FWHM) of the PL peak in region A is only 40 meV, which is comparable to high‐quality WS_2_ samples by mechanical exfoliation.^[^ ^47^ ^]^ Figure 2f demonstrates the optical image, Raman maps of $E_{2g}^{1}$ (351 cm^−1^), *A*_1*g*_ (416 cm^−1^) peaks, and PL maps at 1.97 eV (633 nm) of a single WS_2_ flake; the uniform color and signal indicate the 1L feature and uniform crystallinity of the WS_2_ flake.^[^ ^36^, ^46^ ^]^ The thickness of WS_2_ flake (Figure 2g) measured by atomic force microscopy (AFM) is ≈0.7 nm, corresponding to the reported results of 1L WS_2_.^[^ ^20^, ^32^, ^33^ ^]^ Moreover, the WS_2_ flakes are transferred to the copper grid for scanning transmission electron microscopy (STEM) observation (Section S7, Supporting Information); a well‐organized honeycomb structure of 1L WS_2_ is demonstrated, where the brighter dots correspond to the heavier W atoms and the darker ones correspond to the lighter S atoms (Figure 2h and Figure S7d, Supporting Information). No defects or distortion were observed. The above results indicate the uniform thickness and excellent crystallinity of the resultant 1L WS_2_ plates grown by MSCS.

### 2.3 Domain Size Modulation and Large Area Growth of 1L WS_2_

**Figure 3:**
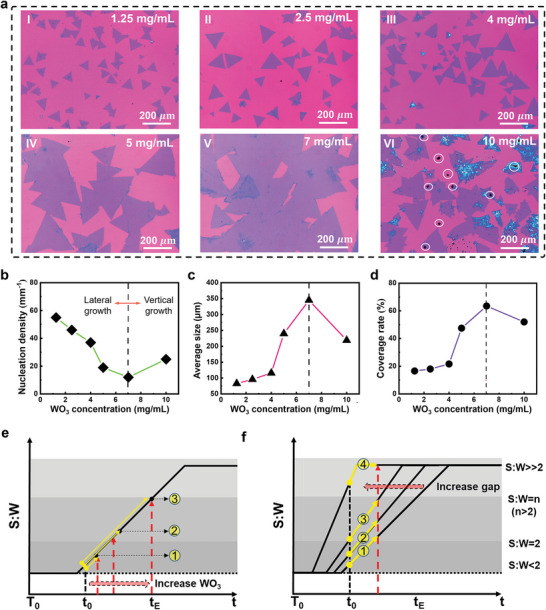
Size modulation of 1L WS_2_ by increasing the concentration of WO_3_. a) Optical images of the crystals grown at different concentrations of WO_3_ for 1.25, 4, 5, 7, and 10 mg mL^−1^ in (I–VI). Inside the white circles in (VI) are residual WO_3_ particles. b–d) Nucleation density, average domain size, and coverage rate of WS_2_ as a function of WO_3_ concentration. e,f) Schematic diagram of WS_2_ growth with respectively appropriate dosage of WO_3_ and different distances between two aspectant substrates.

We further verify that by tuning the concentration of WO_3_ suspension, MSCS can regulate the size of 1L WS_2_ plates and achieve the growth of large‐sized single domain and even centimeter‐scale WS_2_ films. According to the SW‐T diagram (Figure 1e), it is deduced that the concentration of WO_3_ can regulate the dwelling time of the lateral growth. To verify this point, six groups of experiments at different concentrations of WO_3_ (1.25, 2.5, 4, 5, 7, and 10 mg mL^−1^) are carried out. **Figure** **3**a–d shows the optical images of the as‐grown samples and their statistic results of domain size, nucleation density, and coverage rate of WS_2_ flakes versus the concentration of WO_3_. It is found that the gradually increasing size and coverage of 1L WS_2_ flakes can be facially achieved by increasing the concentration of WO_3_. The single WS_2_ flakes with large grain size (average domain size of ≈350 µm), can be fabricated at the optimist WO_3_ concentration of 7 mg mL^−1^ (Figure 3a(V),c). After this, the size of WS_2_ flakes is largely suppressed and the morphology becomes irregular when WO_3_ concentration is increased to 10 mg mL^−1^; some multilayer growths appear with dark blue color (Figure 3a(VI)). Based on Figure 3a–c, the change of nucleation density and coverage rate to WO_3_ concentration is opposite to that of average size of WS_2_ flakes to WO_3_ concentration.

The above growth results on the concentration of WO_3_ can be well‐explained by SW‐T diagram. When the amount of WO_3_ in the unit area is increased, the GR S:W is raised from S:W less than 2 to >>2 (Figure 3e); the WS_2_ flakes grow much more laterally and thus possess bigger domain size. For small amount of WO_3_ (1.25–4.00 mg mL^−1^), only GM‐1L‐S is performed. The triangle WS_2_ flakes with small size are obtained due to slow and short time of lateral growth (Figure 3a(I–III), which are correlated to GR1 (Figure 3e). For the moderate amount of WO_3_ (5–7 mg mL^−1^), GMs‐1L‐S and 1L‐F are performed. The lateral growth of WS_2_ flakes can occur faster and longer which results in bigger size domains (Figure 3a(IV)); they are correlated to GR2 (Figure 3e). More supply of W can be obtained from the liquid precursor when the flakes grow up. The resultant low nucleation density and big‐sized domains further cause the coalesce of WS_2_ flakes as a continuous film (Figure 3a(V)). Consequently, sudden slope variation of nucleation density, average size, and coverage rate of WS_2_ flakes versus WO_3_ concentration is occurred for the moderate amount of WO_3_ (Figure 3b–d). When a large amount of WO_3_ is implemented (10 mg mL^−1^), the resultant flakes are grown laterally and vertically (Figure 3a(VI)); they are correlated to GR3 (Figure 3e).

It is noted that the high concentration of WO_3_ tends to aggregate to big‐sized particles on the substrate (>10 µm, Figure S8a–d, Supporting Information) and thus cannot be totally melted due to the uneven mixing with NaCl. In this case, the unmelted WO_3_ particles do not participate in the growth of WS_2_ plates and some of them finally stick to the upper substrates (marked by dark color in Figure 3a(VI)). Therefore, the locally uneven supply of W source causes the chaotic growth of WS_2_ plates with irregular shapes and partial multi‐layers (dark blue parts in Figure 3a(VI)). The resultant reduced size and coverage by increased nucleation density of WS_2_ plates are attributed to highly distorted GR3 as shown in Figure S8e, Supporting Information. As discussed in Section S8, Supporting Information, the WS_2_ flakes cause the inefficient lateral growth and a fast deposition occurred for the high S:W >> 2 in the late stage of growth. In addition, increasing the gap of confined space (the two aspectant substrates) can also bring evident change to the size of as‐grown WS_2_ flakes but with more multilayer growths (Figure S9, Supporting Information). This can be understood according to the SW‐T diagram as follows: as the gap increases, the S:W curve shifts to the left and thus the GR moves upwards. The corresponding growth of WS_2_ experiences less lateral growth and more vertical growth (Figure 3f, Section S9, Supporting Information).

To demonstrate the potential of MSCS for large area growth of 1L WS_2_, a centimeter‐sized product is synthesized on both SiO_2_/Si substrate and *c*‐plane sapphire (Figure S10, Supporting Information). It is obvious that continuous films of WS_2_ flakes (light purple area on SiO_2_/Si substrate and light white area on *c*‐plane sapphire) are visible in most area of the substrate. They have a uniform thickness upon the entire sample, though there are some additional 2L growths due to locally uneven S:W ratio. Moreover, single domains of 1L WS_2_ on SiO_2_/Si substrate and *c*‐plane sapphire demonstrate maximum size of ≈472 and 630 µm, respectively, which rank them at the top level among recent results (Table S1, Supporting Information). On the contrary, the WS_2_ sample fabricated by CCVD under the same reaction parameters is composed of high density WS_2_ flakes with non‐uniform thickness and limited domain size (Figure S6a, Supporting Information). The above results verify the powerful tunability of MSCS to the growth kinetics for domain size modulation and large area growth of 1L WS_2_.

### 2.4 Layer Number Control of Grown WS_2_ Plates

**Figure 4:**
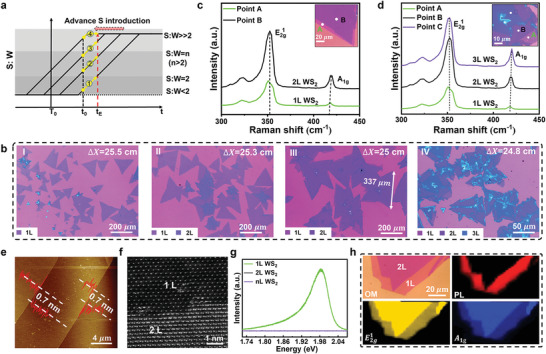
Controllable growth and characterizations of 2L WS_2_. a) SW‐T diagram of controlling layer number of grown WS_2_. b) Optical image of as‐grown WS_2_ sample on SiO_2_/Si at different locations (*∆X* = 25.5, 25.3, 25, and 24.8 cm) from S source, wherein *∆X* depicts the distance between S source and substrate. The color blocks at the lower left corner help to identify the distribution of samples at different layers. c) Raman spectra of 2L WS_2_ (Point B). The inset image belongs to the 2L WS_2_ sample with the step of 1L‐2L on SiO_2_/Si. d) Raman spectra of 3L WS_2_ (Point C). The inset image belongs to the 3L WS_2_ sample with the steps of 1L‐2L‐3L on SiO_2_/Si. e) AFM image of the edge of WS_2_ 2L flake. The inset shows the thickness of the crystal and its corresponding profile. f) HAADF‐STEM image of as‐grown WS_2_ flake for the step of 1L‐2L. h) Optical image of 2L WS_2_ crystal, and the corresponding PL Mapping (A exciton) and Raman ($E_{2g}^{1}$ and *A*_1*g*_). g) PL spectra of 1L, 2L, and *n*L WS_2_ (Points A–C). h) Optical image of 2L WS_2_ crystal, and the corresponding PL mapping (A exciton) and Raman ($E_{2g}^{1}$ and *A*_1*g*_).

According to Figure 1e, when the 2L growth is progressed, 2L WS_2_ plates are created as the GR passes the S:W zone of *n* (*n* > 2). This condition can be reached by using a properly‐elevated concentration of WO_3_ (GR3 in Figure 3e), increasing the gap of two aspectant substrates (GR3 in Figure 3f) or by advancing the introduction of S vapor (GR3 in **Figure** **4**a). However, we failed to obtain the desired 2L products in practice by the former two strategies as shown in Figure 3a(VI) and Figure S9, Supporting Information, due to the short dwelling time of GR at S:W ≈ *n* (Sections S8 and S9, Supporting Information). The latter strategy works for the selectively grown 1L/2L WS_2_ plates by decreasing *∆X* (the distance between S source and the substrate is fixed at the center of furnace). Figure 4b shows the gradual evolution of WS_2_ plates from 1L to 2L at lower *∆X*. The color of WS_2_ plates varies according to their layer number upon slightly changed illumination; based on the color contrast, it is convenient to identify the layer number of WS_2_ samples. It is found that 1L WS_2_ plates are the dominant product at *∆X* = 25.5 cm (Figure 4b(I)). When *∆X* is decreased to 25.3 cm, a mixed 1L and 2L sample is obtained (Figure 4b(II)). Further decrease of *∆X* to 25 cm results in the majority of 2L WS_2_ plates with the average domain size of ≈300 µm (Figure 4b(III),c), which is the largest value among the previously reported works (summarized in Table S2, Supporting Information). When *∆X* is decreased to 24.8 cm, uneven 3L and multi‐layered WS_2_ plates appear (Figure 4a(IV)), which is also identified by Raman spectra in Figure 4d. In fact, at the smaller *∆X*, the shape of WS_2_ plates is more irregular; this result indicates a relatively fast but unstable growth kinetics.

Besides centimeter scale of 2L WS_2_ film on SiO_2_/Si substrate, we obtain 2L WS_2_ film on *c*‐sapphire using the same reaction conditions by MSCS strategy (Figure S11, Supporting Information); this proves the great potential of the introduced MSCS strategy to fabricate large area 2L WS_2_ films on different kinds of substrates. However, under the same growth conditions for 2L WS_2_, the prepared WS_2_ samples (Figure S6b, Supporting Information) by CCVD method are irregular crystals with high nucleation density and uncontrollable film thickness; this verifies the controllability of MSCS strategy in regulating the growth of WS_2_ with more layer number.

The selective growth of the above 1L and 2L WS_2_ plates by MSCS can be explained as below. The sulfur source is placed in the upstream and its evaporation depends on the distance from the center of furnace (*∆X*). Different *∆X* indicates various introduction time of S vapor to the substrate. For such a growth of WS_2_ plates, we fix the amount of WO_3_ at a moderate concentration of 5 mg mL^−1^ to make sure the sufficient melting of WO_3_ at the reaction temperature under the assistance of NaCl. As reported before, a moderate concentration ratio of precursor elements (S:W molar ratio) is an important factor driving the controlled layer number of grown WS_2_.^[^ ^18^, ^48^ ^]^ Here, when *∆X* decreases from 25.5 to 24.8 cm, the curve of S:W shifts to the left and thus enables GR to pass the S:W zones from ≈2 to *n* and even larger values (Figure 4a). Consequently, the controllable growth of 1L/2L WS_2_ plates is achieved. Moreover, the above results suggest that, by precisely tuning the introduction time of S vapor, it is possible to selectively fabricate *n*‐layer (*n* > 2) WS_2_ plates. As shown in Figure 4b(IV), small 3L WS_2_ plates are fabricated when *∆X* = 24.8 cm, while the thickness distribution is not uniform, the morphology is irregular, and the experimental repeatability is poor. This is due to the abrupt temperature gradient across furnace mouth, where S vapor is dramatically increased with a further tiny decrease of *∆X* (Section S12, Supporting Information). This situation brings challenges to the growth of uniform and large‐sized WS_2_ plates of 3L and above. The controlled layer number growth of TMDCs may be achieved by this MSCS strategy with modified furnacing setups that can gradually change S vapor with *∆X*. We also successfully selectively fabricated 2L MoS_2_ with similar MSCS strategy (Figure S13, Section S12, Supporting Information), indicating the generality and validity of our proposed X:M‐T growth diagram for the growth of other 2D TMDCs.

The as‐grown 2L WS_2_ plates are comprehensively characterized as follow. Raman spectra (Figure 4c) of point A shows a typical WS_2_ monolayer structure with ≈65.5 cm^−1^ difference between $E_{2g}^{1}$ mode and A_1g_ mode, while point B of 2L region shows an enhanced Raman intensity and higher wave number difference between $E_{2g}^{1}$ mode and A_1g_ mode (66 cm^−1^). Apart from 2L WS_2_, 3L WS_2_ shows a larger difference (67 cm^−1^) between $E_{2g}^{1}$ mode and A_1g_ mode (Figure 4d). Figure 4e presents the AFM image of the stepped 2L WS_2_ plate, where the line‐scanning from the substrate to the sample's top surface shows two single steps of 0.7 nm, indicating the characteristic of 1L‐2L for WS_2_.^[^ ^34^ ^]^ In addition, Figure S14a, Supporting Information, reveals the thickness of ≈1.5 nm for the WS_2_ sample, further confirming the 2L feature of the as‐grown WS_2_ film.^[^ ^18^ ^]^ Figure 4f and Figure S14b–d, Supporting Information, show the HADDF‐STEM image of the stepped area of 1L−2L WS_2_ plate; the 1L region displays a classic structure of 1L WS_2_ as that in Figure 2h. The 2L region has a typical 3R phase structure of 2L WS_2_, where extra W atoms reside on S atoms. It can be also found that the 1L/2L interface is not smooth, this suggests a fast and unstable dynamic growth of the second layer on the first layer. Figure 4g shows the typical PL spectra of 1L and 2/*n*L (*n* > 2) WS_2_. The 1L plate shows a strong PL emission at 630 nm (1.97 eV), while 2/*n*L (*n* > 2) plate presents a greatly‐suppressed PL emission due to the indirect band gap characteristics of multilayered WS_2_. Figure 4h demonstrates the optical, PL and Raman mapping images, all of which exhibiting a uniform contrast in 1L and 2L areas. Based on these characterization results, it is concluded that the as‐grown 2L WS_2_ plates have a uniform structure and good quality.

### 2.5 Controllable Growth of MoS_2_‐WS_2_ Lateral/Vertical Heterostructures

**Figure 5:**
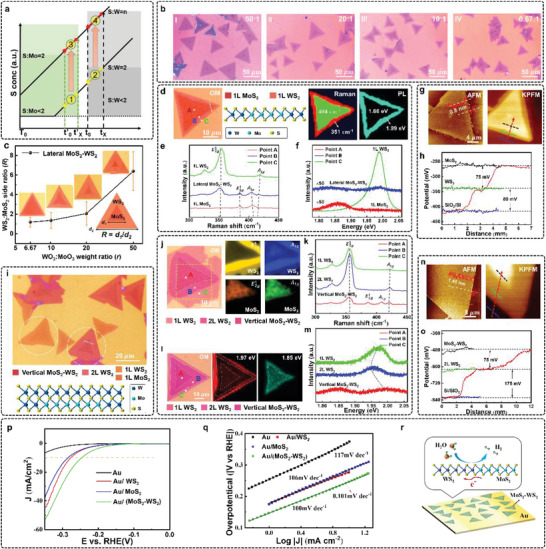
Characterizations of MoS_2_‐WS_2_ lateral/vertical heterostructures. a) Schematic diagram of MoS_2_‐WS_2_ lateral heterostructures growth with appropriate reaction time. b) Optical images of the MoS_2_‐WS_2_ lateral heterostructures with four different WO_3_:MoO_3_ weight ratios (*r*) of 6.67, 10, 20, and 50. c) The side ratio *R* with a function of *r* from 6.67 to 50. The inset images are corresponding MoS_2_‐WS_2_ lateral heterostructures and the definition of size ratio (R) of WS_2_ to MoS_2_. d) The optical image of MoS_2_‐WS_2_ lateral heterostructures and corresponding Raman and PL mapping. e,f) PL and Raman spectra of point A (MoS_2_), point B (WS_2_) and point C (interface of the heterostructure) in (d). g) AFM image and Kelvin probe force microscope (KPFM) images for MoS_2_‐WS_2_ lateral heterostructures. It is noted that the KPFM image is consistent with the AFM image. h) Plots of the surface potential along four dash‐lines depicted in (g). i) Optical image of the MoS_2_‐WS_2_ heterostructures. The flakes in the white dotted box are MoS_2_‐WS_2_ vertical heterostructures. The bottom inset is a schematic diagram of MoS_2_‐WS_2_ vertical heterojunction, where a monolayer MoS_2_ is covered by WS_2_. j) The optical image of MoS_2_‐WS_2_ vertical heterostructures and its corresponding Raman mapping. k) Raman spectra of point A (MoS_2_‐WS_2_ vertical heterostructures), point B (2L WS_2_) and point C (1L WS_2_) in (j). l) The optical image of MoS_2_‐WS_2_ vertical heterostructures and the corresponding PL mapping. m) PL spectra of point A (MoS_2_‐WS_2_ vertical heterostructures), point B (2L WS_2_) and point C (1L WS_2_) in (i). n) AFM image and corresponding KPFM images of MoS_2_‐WS_2_ vertical heterostructures. o) Plots of the surface potential along the four dash‐lines depicted in (n). p) HER polarizing and q)Tafel slope curves for supporting Au electrode, WS_2_, MoS_2_, and MoS_2_‐WS_2_ lateral heterostructures. r) Schematic of charge transfer promoting HER process.

The MSCS method can be further implemented for the growth of MoS_2_‐WS_2_ lateral/vertical heterostructures based on a coupled S:Mo and S:W‐T growth diagram. **Figure** **5**a shows the designed GRs for this purpose; it consists of two chronological growth stages for 1L MoS_2_ and WS_2_ plates (GRs12 and GRs34). The required experiment for the GR 12 lateral heterostructure is conducted by using the mixed metal precursors (WO_3_ and MoO_3_ with NaCl). Figure 5b shows the optical images of the as‐grown MoS_2_‐WS_2_ lateral heterostructures with different *R*s, where MoS_2_ triangles (dark purple) are laterally surrounded by WS_2_ (light purple). It is noted that the side ratio of WS_2_ to MoS_2_ in the lateral heterostructures can be fully modulated (≈1–8) by *r* (Figure 5b,c) at a fixed WO_3_ concentration of 5 mg mL^−1^. For a large ratio of 50:1 for WO_3_:MoO_3_, a relatively broad WS_2_ frame and tiny MoS_2_ core are obtained in single plates. For a small ratio of WO_3_:MoO_3_ (6.67:1), the WS_2_ frame greatly shrinks and the MoS_2_ core expands; this result is similar to a previous report.^[^ ^51^ ^]^ The growth of 1L MoS_2_‐WS_2_ lateral heterostructures can be well understood by considering different melting points of MoO_3_ and WO_3_ (≈750 °C for MoO_3_ and ≈800 °C for WO_3_ with the help of NaCl flux agent).^[^ ^43^ ^]^ At first, MoS_2_ nucleates and grows as 1L plates (GR1), then, WS_2_ takes a laterally epitaxial growth to the MoS_2_ core (GR2). The whole growth process of 1L MoS_2_‐WS_2_ lateral heterostructure follows the designed GR12 in Figure 5a very well. On the contrary, the as‐grown sample of CCVD‐base with similar reaction parameters is composed of complicated components (Figure S6c,d, Section S4, Supporting Information) and uneven layer number. The above growth observations verify the effective tunability of MSCS strategy for MoS_2_‐WS_2_ lateral heterostructures. Therefore, by deliberately arranging different metal precursors, it is expected to design and achieve multicomponent 2D lateral heterostructures by MSCS in the near future. In addition, it is noted that the size of as‐grown MoS_2_‐WS_2_ lateral heterostructures (up to 50 µm) is smaller than that of 1/2L WS_2_ (up to 300 µm, Figures 3 and 4). This can be understood by considering that NaCl was consumed by the growth of MoS_2_ plates and the remained amount of NaCl was far from the optimized value for large sized growth of WS_2_, leading to relatively small sized products.

The above 1L MoS_2_‐WS_2_ lateral heterostructures are verified and characterized as below. The optical image, Raman, and PL mapping of a single triangle plate (Figure 5d) display a clear contrast between the core and frame regions. The Raman maps are taken at 404 cm^−1^ (*A*_1_ *_g_* mode of MoS_2_, green) and 351cm^−1^ ($E_{2g}^{1}$ mode of WS_2_, red), and PL mapping at 1.86 eV (660 nm of MoS_2_, nearly dark) and 1.97 eV (630 nm of WS_2_, cyan).^[^ ^49^, ^50^ ^]^ The Raman (Figure 5e) and PL (Figure 5f) spectra of points A–C in Figure 5d further show characteristic peaks of the core, interface, and frame regions of 1L MoS_2_ and WS_2_. The atomic structure model of 1L WS_2_‐1L MoS_2_‐1LWS_2_ corresponded to the white dotted line in the OM image is given in Figure 5d. It is noted that, the PL peak of MoS_2_ in 1.86 eV is 150 times weaker than MoS_2_, similar to the previous reports on MoS_2_‐WS_2_ lateral heterostructures.^[^ ^50^, ^51^ ^]^ The much weaker PL emission of MoS_2_ in MoS_2_‐WS_2_ lateral heterostructures may be attributed to the charge transfer from MoS_2_ to WS_2_, which is verified by the following charge transfer results.

The AFM result (Figure 5g) reveals a uniform thickness of ≈0.8 nm from the center to the edge of flake, indicating the 1L feature of MoS_2_‐WS_2_ lateral heterostructures.^[^ ^50^, ^52^, ^53^, ^54^ ^]^ In contrast to the weak contrast of topography image (left panel of Figure 5g), surface potential mapping (right panel of Figure 5g), recorded by Kelvin probe force microscope (KPFM), supplies a sharp contrast at the interface of MoS_2_ and WS_2_, similar to those obtained in Figure 5d. Figure 5h shows the line scanning results of surface potential at the regions of MoS_2_, WS_2_, substrate, and their entire regions. The flat potential curves recorded at the MoS_2_ and WS_2_ domains indicate a uniform quality. In addition, a surface potential difference of ≈80 mV between WS_2_ and MoS_2_ is measured, which is consistent with the previous reports.^[^ ^50^, ^52^ ^]^ Due to different surface potentials of MoS_2_, WS_2_, and substrate, a stair‐stepping potential curve is obtained across the specified regions along red line in Figure 5g. This property results in the photo‐generated charge transfer between them and a suppressed PL emission of MoS_2_.^[^ ^52^ ^]^

Compared to the 2D TMDCs‐based lateral heterostructures, the vertical heterostructures are more attractive due to plane‐to‐plane contact of individual 2D TMDCs layers with unique physical properties; these guarantee numerous applications.^[^ ^50^, ^53^, ^55^, ^56^, ^57^ ^]^ Referring to SW‐T diagram of Figure 5a, the growth of MoS_2_‐WS_2_ vertical heterostructure can be achieved by GRs34, where GRs12 is boosted to GRs34 by increasing the concentration of S vapor through shortening *∆X* (for details refer to Experimental Section). Accordingly, small pieces of 1L triangle plates of MoS_2_ are formed first (GM‐1L‐F at GR3 with S: Mo ≈ 2). Then, WS_2_ takes an epitaxial growth to the pregrown MoS_2_ core laterally and vertically (GM‐2L at GR4 with S:W ≈ *n*) to form a 2L WS_2_‐$\frac{1L\;{\mathrm{WS}}_{2}}{1L\;{\mathrm{MoS}}_{2}}$‐2L WS_2_ structure. Figure 5i shows the typical samples obtained by GR13, where the vertical and lateral heterostructures are mixed. The mixed products here can be attributed to the uneven mixing of WO_3_ and NaCl, which makes the local S:W deviate from the intended design in some sites on the substrate. It is easy to distinguish the plates containing vertical/lateral heterostructures from optical images as they are 2L with darker color (the dash‐line circled plates in Figure 5i), while the lateral heterostructures are 1L with lighter color (the dotted‐line circled plates in Figure 5i). The white dotted line on the vertical heterostructure at the bottom of Figure 5i is corresponded to the atomic structure model of 2L vertical heterostructure. We further perform the following comprehensive characterizations to verify the vertical heterostructure of the as‐grown sample. The Raman and PL spectra and mapping indicate the presence of both 1L MoS_2_ and 1L WS_2_ at the core of sample while 2L WS_2_ is appeared at the frame (Figure 5j–m, Section S14, Supporting Information). It is also found that PL emissions of the region (point C) on the MoS_2_‐WS_2_ vertical heterostructure are strongly suppressed; this can be attributed to their 2L features and the enhanced charge transfer from MoS_2_ to WS_2_ for their much larger plane‐to‐plane contact. ^[^ ^50^, ^51^ ^]^ The AFM image in Figure 5n reveals the thickness of the flake sample by ≈1.5 nm from the edge to center, which confirms the 2L feature of MoS_2_‐WS_2_ vertical heterostructures. Moreover, a sharp potential difference is seen in the corresponding KPFM result between the core and edge of the flake (Figure 5o); this observation further confirms a MoS_2_‐WS_2_ vertical heterostructure in the core of the flake. It is worthy to note that we could not fabricate such vertical heterostructures on a large scale because of their mixed vertical and lateral growth, with relatively poor control and reproducibility compared to the growth of the preceding products. Our research endeavor to improve the controllable fabrication of MoS_2_‐WS_2_ and other multi‐component, muti‐layered vertical heterostructures is in progress.

The 1L MoS_2_‐WS_2_ lateral heterostructure can be repeatedly fabricated in a large scale due to the facile GR12. We used this setup as a model system to study the intrinsic catalytic process and mechanism of heterostructures considering recent great progress on TMDCs for water splitting.^[^ ^57^, ^58^, ^59^, ^60^ ^]^ In the present study, the hydrogen evolution reaction (HER) activity of MoS_2_, WS_2_, and 1L MoS_2_‐WS_2_ lateral heterostructures supported by Au foils is explored in acid condition (0.5 m H_2_SO_4_ solution) and the corresponding polarization curves are depicted in Figure 5p. The MoS_2_‐WS_2_ lateral heterostructures exhibits an overpotential ‐246.1 mV versus RHE at the current density of 10 mA cm^−2^. This value is lower than that of the WS_2_ film (−276.8 mV) and MoS_2_ (−283 mV). Besides, the Tafel slope of 1L MoS_2_‐WS_2_ lateral heterostructures is 100 mV dec^−1^ (Figure 5q), slightly lower than that of the 1L WS_2_ flakes (101.5 mV dec^−1^) and MoS_2_ (107.5 mV dec^−1^). It is thus concluded that the MoS_2_‐WS_2_ lateral heterostructures engineering by hybridizing 2D TMDCs can effectively improve HER activities. The enhanced electrochemical performance may be attributed to the following two aspects: 1) the increased catalytic sites at the interface of MoS_2_ and WS_2_ due to their lattice mismatch, as summarized in previous reports.^[^ ^61^ ^]^ 2) the synergistic effect of electron transfer between components due to their potential difference, as shown in Figure 5i. The electron transfer between MoS_2_ and WS_2_ has been verified by the staged potential curve shown in Figure 5h,r. This simple and pure model system of MoS_2_‐WS_2_ lateral heterostructures substantially helps to discover the mechanism of the enhanced HER performance of hybrid TMDCs catalysts by excluding extrinsic factors.^[^ ^58^ ^]^

## 3 Conclusion

Thanks to the mild growth kinetics offered by MSCS, an effective lateral/vertical CVD growth can be facilely created and regulated via proper S:W zones corresponded to greatly broadened time windows. We proposed a SW‐T growth diagram as a mapping guide for a general understanding of the CVD growth of 2D WS_2_, by which the growth processes can be descripted, and their products can be predicted. Referring to the SW‐T diagram, GRs for desired 2D WS_2_ were facilely designed and performed based on the MSCS, leading to the comprehensive CVD growth management of atomically thin WS_2_ plates, including the versatile growth control of domain size, layer number, and lateral/vertical heterostructures. The lateral heterostructures showed an enhanced hydrogen evolution reaction (HER) performance. Overall, this work highlighted the importance of S:W in the CVD method with an SW‐T diagram as an unprecedented map and practical guide for the growth design and control of WS_2_ plates. We anticipate that the substantially enriched understanding of CVD growth kinetics and the innovative application of X:M‐T growth diagram would effectively and scientifically pave the way for the growth management of *all* 2D TMDCs.

## 4 Experimental Section

### Precursor Preparation

WO_3_ suspension: To obtain certain weight ratio of WO_3_/NaCl suspension (5:1, 5:2, 2:1, 3:2), the dosage of NaCl nanoparticles was calculated and added to the WO_3_ suspension. The dosages of DI water and WO_3_ in all experiments were fixed as 4 mL and 20 mg, respectively. The dosage of NaCl for four sets of experiments with certain weight ratios of WO_3_/NaCl (5:1, 5:2, 2:1, and 3:2) was 4, 8, 10, and 13.5 mg, respectively (see Table S3, Supporting Information, for details). Different concentrations of WO_3_ with an optimized weight ratio (2:1) of WO_3_/NaCl were tabulated in Table S4, Supporting Information, and the optical images of WO_3_ suspension were shown in Figure S1a, Supporting Information.

MoO_3_/NaCl suspension: The dosages of DI water, NaCl were fixed as 4 mL, 10 mg, and MoO_3_ were given range of 0.4–0.8 mg, respectively.

WO_3_/MoO_3_/NaCl suspension: To obtain certain weight ratio for WO_3_/MoO_3_/NaCl suspension (20:3, 10:1, 20:1, 50:1), the dosage of MoO_3_ nanoparticles (99.9%, Mackin) were calculated and added to the WO_3_ suspension. The dosages of DI water, NaCl and WO_3_ in all experiments were fixed as 4 mL, 10 mg, 20 mg, respectively. The dosage of MoO_3_ for four sets of experiments with certain *r* (20:3, 10:1, 20:1, 50:1) was 3, 2, 1, and 0.4 mg, respectively (see Table S5, Supporting Information for details).

### MSCS‐CVD Growth of WS_2_

S power (500 mg, 99.998%, Sigma‐Aldrich, USA) was placed at the upstream edge of the furnace. The SiO_2_/Si substrate spin‐coated with WO_3_ suspension was placed at the center of furnace. Another piece of clean Si/SiO_2_ substrate was put face down above the previous Si/SiO_2_ substrate to form a confined space. The system was heated up to 840 °C in 50 min and then maintained in the same temperature for 5 min and finally naturally cooled down to room temperature. During the whole process, Ar flow (40 sccm) was used as the carrier gas. The reactions were conducted under atmospheric pressure. For the experiments of as‐grown WS_2_ sample in Figure 2, the WO_3_ concentration was set as 5 mg mL^−1^ (as shown in Table S4, Supporting Information). For the size modulation of 1L WS_2_ in Figure 3, the corresponding WO_3_ suspension parameters are shown in Table S4, Supporting Information. For the growth of 1L WS_2_, the distance between S source and substrate (*∆X*) was fixed at ≈25.5 cm, while for the growth of 2L WS_2_, it was *∆X* = 25 cm.

### MSCS‐CVD Growth of MoS_2_

The reactions were conducted under atmospheric pressure. The concentration of MoO_3_ suspension was set as 2 mg mL^−1^ with NaCl:MoO_3_ = 1:2 (mass ratio). 1L/2L MoS_2_ can also be successfully fabricated by MSCS strategy. For 1L MoS_2_, ∆X was fixed as ≈24.5 cm to ensure that the Mo source was just around 700 °C when the S powder melted. Different form 1L MoS_2_, 2L MoS_2_ can be fabricated with lower ∆X (≈22.5.cm). The system was heated up to 800 °C in 50 min, maintained in the same temperature for 8 min and then naturally cooled down to room temperature. During the whole CVD process, Ar (40 sccm) flow was used as the carrier gas.

### MSCS CVD Growth of MoS_2_‐WS_2_ Heterostructures

The SiO_2_/Si substrate, spin‐coated with the mixture of WO_3_ and MoO_3_ suspension, was placed at the center of furnace. Another piece of clean SiO_2_/Si substrate was put face down above the bottom Si/SiO_2_ substrate to form a confined space. *∆X* was fixed at 25.0 cm. The system was heated up to 800 °C at the ramping rate of 15 °C min^−1^ and then gradually heated up to 830 °C in 5 min and maintained in the same temperature for 3 min, and finally naturally cooled down to room temperature. During the whole process, Ar flow (40 sccm) was used as the carrier gas. The reactions were conducted under atmospheric pressure. For the side ratio modulation of MoS_2_‐WS_2_, the corresponding WO_3_/MoO_3_ suspension parameters are shown in Table S5, Supporting Information. For the vertical growth of MoS_2_‐WS_2_ heterostructures, the distance between S source and substrate (*∆X*) was fixed at ≈24.8 cm; the dosage of WO_3_, MoO_3_, NaCl, and DI water was 20 mg, 1 mg, 10 mg and 4 mL, respectively.

### COMSOL Multi‐Physics Simulation

Since the flow velocity in the tubular furnace is relatively flat, the coupling of laminar flow and dilute material transfer modules is selected to have a balanced concentration distribution of S vapor on the substrate surface in the CVD reaction. In order to simplify the computation, we construct a simple quartz tube model as shown in Figure S3a, Supporting Information. It is worth noting that, in the real experiment, the gap between the two substrates is close to zero. In our simulation, we set the gap between the two substrates as 0.7 mm for the calculation and accuracy in the simulation process. The S concentration at the inlet is 1 mol m^−3^ (which can be regarded as a normalized unit concentration), and the gas flow rate of 40 sccm.

### Preparation of STEM Sample

As shown in Figure S7, Supporting Information, a thin poly (methyl methacrylate) (PMMA, A4 950 K in anisole, Micro Chem) layer was spin‐coated (4000 rpm, 50 s) on the as‐grown WS_2_ sample and baked at 200 °C for 5 min. Then, the WS_2_ sample was put in KOH (2 m) solution for nearly 30 min in 60 °C until the WS_2_ sample was separated form SiO_2_/Si substrate. The PMMA/WS_2_ film was gently picked up with a clean substrate and placed floating in DI water several times before being fished by a copper grid. The copper grid with WS_2_/PMMA was soaked in acetone for 6–8 h until the PMMA layer was removed, and then transferred to DI water to remove acetone. Finally, the copper grid was dried up under heating lamp for nearly 5 min.

### Characterizations

An Olympus optical microscope (BX 51) equipped with a charge‐coupled device camera was used to capture the optical images. An AFM machine (Bruker, Multimode 8) was utilized to scan the heights and the surface potential of the WS_2_ and MoS_2_ crystals under ScanAsyst‐Air mode and KPFM mode. KPFM mode used tapping amplitude modulation mode, where the potential drives routing on the tip. In this mode, a higher potential represented a lower work function. Raman and PL measurements for point spectra and mapping were performed on a commercial system (Horiba, LabRAM HR Evolution) using a 532‐nm laser ray for excitation. The laser with a power of 4.25 µW was focused on the sample by a 50× NA 0.95 objective. A JEOL JEM ARM 200CF instrument operated at 60 kV was used to obtain the STEM images. The crystal structure of WO_3_/NaCl mixtures and residues were determined by X‐ray diffraction (XRD) on a D8 ADVANCE ECO (Bruker) X‐ray diffractometer, where the wavelength of generated X‐ray was 1.5418 Å (Cu K*α*, isolated with a Ni foil filter).

### Electrochemical Test

Electrochemical measurements were carried out in a conventional three‐electrode electrochemical cell using a CHI660D electrochemical workstation (CHI Instruments Inc., Shanghai, China). Hg/HgO and graphite were used as the reference electrode and the counter electrode, respectively. The MoS_2_‐WS_2_ lateral heterostructures supported by Au foil with a dimension of 0.5 cm × 0.5 cm served as the working electrode. The catalytic performance was measured using linear sweep voltammetry versus reversible hydrogen electrode (RHE) with a scan rate of 5 mV s^−1^ in 0.5 m H_2_SO_4_ (pH = 0).

### Statistical Analysis

All statistical analyses, unless stated, otherwise, were performed with Origin 9.1. The data obtained from Raman, AFM, KPFM, PL, were the original data without normalization. For simulations of S:W evolution in open/confined spaces, the S concentration was normalized to [0, 1] (Figure 1d). For coverage rate (*w*) in this manuscript, which was defined as $\frac{S_{\mathrm{flakes}}}{S_{\mathrm{total}}}$, where *S*_flakes_ is the area of the film in the OM, *S*_total_ is the area of the entire optical image, is directly obtained by Photoshop CS6. Nucleation density *ρ* = samples size (*n*_flakes_) per optical image/image area, which is calculated by Office Excel 2019 software. The shape of WS_2_ crystal grown from MSCS CVD was almost triangular which could be thought of as a regular triangle. Therefore, domain size(*a*) satisfied the following relation: *S*_flakes_ = *n*_flakes_ $\frac{\sqrt{3}}{4}\;a^{2}$.^[^ ^44^ ^]^ Standard deviation (SD) of domain size (*a*) is obtained by formula: $\mathrm{SD}\;=\sqrt{\frac{\sum_{i=1}^{n}{\left(a_{i}-\bar{a}\right)}^{2}}{n-1}}\;$, where “*n*” for each statistical analysis is equal to the number of flakes on etch OM. The SD of domain size in Figure 2e here, SD_region A_ = 17.9, SD_region B_ = 27.38, SD_region C_ = 3.16. Corresponding to different concentrations (1.25, 2.5, 4, 5, 7, and 10 mg mL^−1^) of samples (Figure 3c), the SDs can be caculated as SD_1.25_ = 11.59, SD_2.5_ = 15.07, SD_4_ = 13.92, SD_5_ = 20.21, SD_7_ = 17.6, SD_10_ = 28.5. In Figure S4f, Supporting Information, for different mass ratio of NaCl:WO_3_ (1/5, 2/5, 1/2, and 2/3), the corresponding SD_1/5_ = 5.7, SD_2/5_ = 32.04, SD_1/2_ = 25.48, SD_2/3_ = 13.44.

## Conflict of Interest

The authors declare no conflict of interest.

## Author Contributions

W.Q. conducted most of the experiments and wrote the manuscript. W.S. conducted STEM characterizations. J.L. helped with the sample preparation and characterizations of 2L MoS_2._ G.Y. helped with the sample preparation. J.M. helped with the HER tests. S.R. gave suggestions. A.A. revised the manuscript. C.C. supervised the whole process.

## Supporting information

Supporting Information

Supporting Information

Supporting Information

Supporting Information

Supporting Information

## Data Availability

Research data are not shared.
