# Supplementary material for: Modified Spatially Confined Strategy Enabled Mild Growth Kinetics for Facile Growth Management of Atomically‐Thin Tungsten Disulfides
Source: Adv Sci (Weinh). 2022 Nov 29;10(3):2205638. doi: 10.1002/advs.202205638 (PMC9875684; doi:10.1002/advs.202205638)
Supplement: Supplementary file 1 — Supporting Information [file ADVS-10-2205638-s005.pdf]

ADVANCED SCIENCE
Open Access

Supporting Information

for *Adv. Sci.*, DOI 10.1002/advs.202205638

Modified Spatially Confined Strategy Enabled Mild Growth Kinetics for Facile Growth Management of Atomically-Thin Tungsten Disulfides

*Qun Wang, Shi Wang, Jingyi Li, Yichen Gan, Mengtian Jin, Run Shi, Abbas Amini, Ning Wang and Chun Cheng**

Supporting Information

Modified Spatially Confined Strategy Enabled Mild Growth Kinetics for Facile Growth Management of Atomically-Thin Tungsten Disulfides

Qun Wang[a], Shi Wang[c], Jingyi Li[a], Yichen Gan[a], Mengtian Jin[a], Run Shi[a], Abbas Amini[d], Ning Wang[c] and Chun Cheng[a, b*]

[a] Department of Materials Science and Engineering, Southern University of Science and Technology, Shenzhen 518055, P. R. China

[b] Guangdong Provincial Key Laboratory of Energy Materials for Electric Power, Southern University of Science and Technology, Shenzhen 518055, China

[c] Department of Physics and Center for Quantum Materials, Hong Kong University of Science and Technology, Hong Kong, P. R. China

[d] Center for Infrastructure Engineering, Western Sydney University, Kingswood, NSW 2751, Australia

* To whom correspondence should be addressed, *E-mail*: chengc@sustech.edu.cn

S1. Preparation process of MSCS for the growth of 2D WS_2

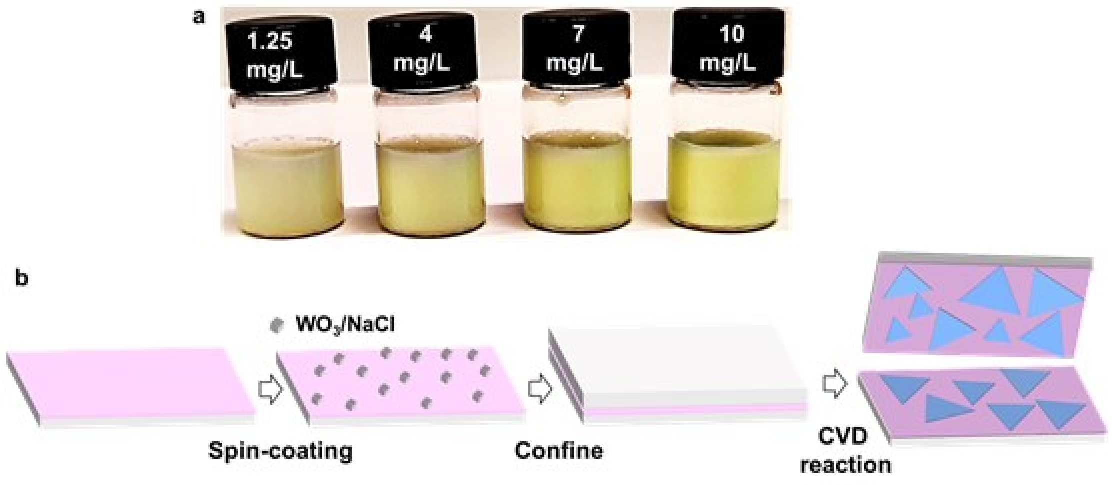

Figure S1. The preparation process for CVD growth of 2D WS_2 by MSCS. (a) The suspension of different concentrations of WO_3 in DI water. A certain amount of NaCl is added to form the mixture of NaCl and WO_3. (b) The sketch map for the 2D WS_2 growth by MSCS: 1) disperse the WO_3 particles on the SiO_2/Si substrate by spin-coating the WO_3/NaCl suspension; 2) form confined space within two aspectant substrates; 3) perform the CVD growth of 2D WS_2.

The preparation and reaction processes for the MSCS growth of 2D TMDCs are simple and accessible.

S2. Reaction of WO_3/NaCl mixture and growth of WS_2 in CVD

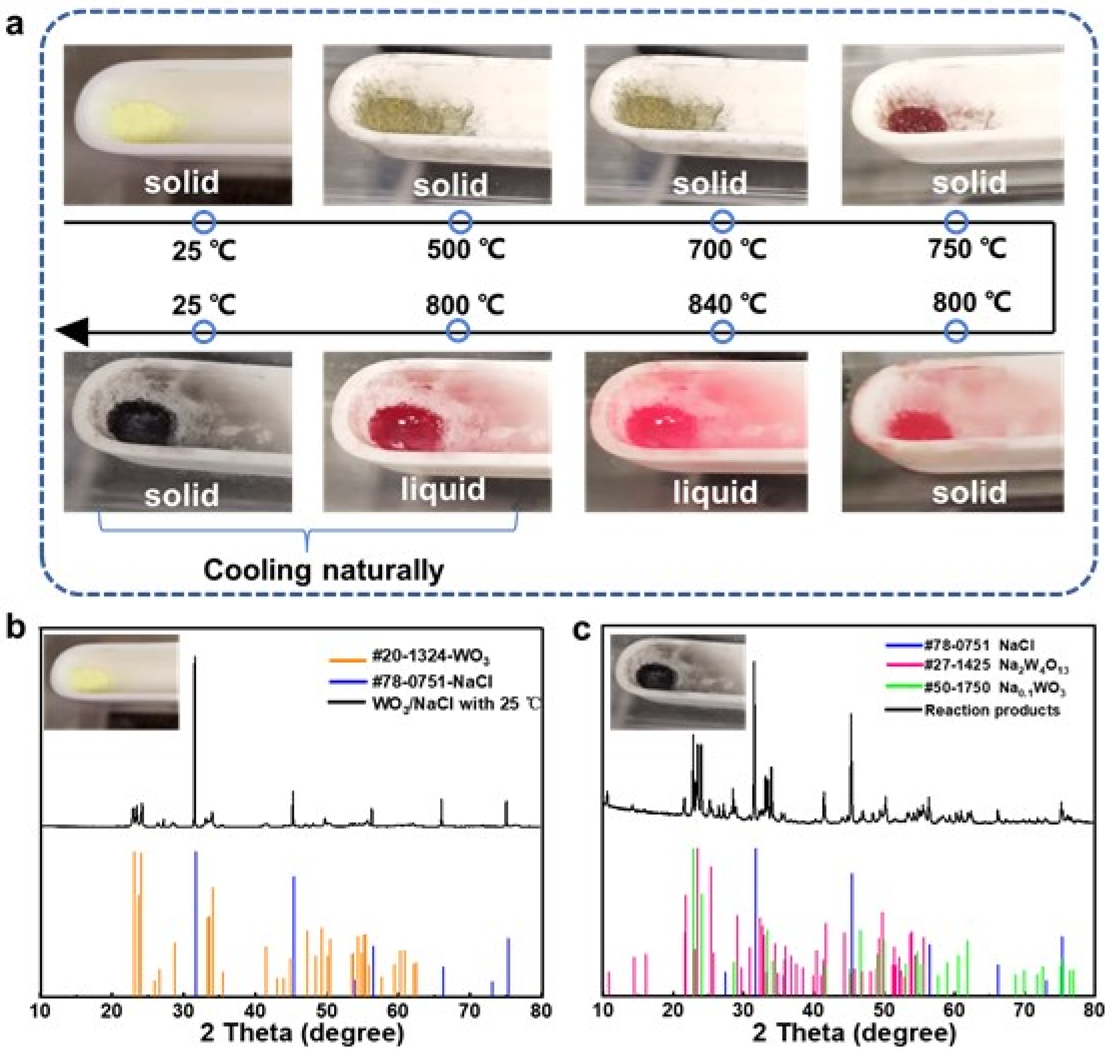

Figure S2. In-situ observation of mixture of WO_3 and NaCl at elevated temperature and residue products. (a) Optical images of 150 mg WO_3/NaCl mixture (with the mass ratio of ~ 2:1 for WO_3: NaCl) in the furnace under 40 sccm Ar when heated to 840 ℃ at the ramping rate of 15 ℃/min and then cooled down naturally. (b) XRD pattern of WO_3/NaCl mixture at 25 ℃. The inset picture is WO_3/NaCl mixture. (c) XRD pattern of the reaction products of WO_3/NaCl mixture when heated to 840 ℃ and then cooled down naturally. The inset picture is the reaction products which contain $Na_2W_2O_{13}$, $Na_{0.1}WO_3$ and residual NaCl.

To study the reaction of WO_3 with NaCl, a typical CVD process without sulfur was performed. As shown in **Figure S2a,** we in-situ observed the melting of mixed

WO_3 and NaCl with the WO_3/NaCl mass ratio of ~2:1 upon increasing temperature. Optical photos were taken at 25 °C, 500 °C, 700 °C, 750 °C, 800 °C, and 840 °C, respectively. According to **Figure S2b** and **c**, the reaction products of WO_3 and NaCl at 840 °C were $Na_2W_2O_{13}$ and $Na_{0.1}WO_3$ in a liquid form.

It was deduced that when a typical CVD was performed, S powder in the upstream of the tubular furnace began to evaporate into the central substrate region at about 800 °C, and reacted with molten Na_xWO_y ($Na_2W_2O_{13}$ and $Na_{0.1}WO_3$) to form WS_2. Also, the white alumina crucible was "stained" at ~800 °C; at this temperature, the vapor phase product (inset of **Figure S2c**) began to release. According to previous reports, these gaseous products are WO_uCl_v ($WOCl_4$, WO_2Cl_2, etc.) [1, 2], which can also react with S vapor to form WS_2. Based on the above analysis, we explain the possible growth process as follows.

During the temperature ramping to the growth temperature of 800-840 °C, the co-sublimated WO_3 and NaCl reacted first as Equation (1).

$$\mathbf{WO_3}\,(s)+\mathbf{NaCl}\,(s)\rightarrow\mathbf{Na_xWO_y}\,(l)+\mathbf{WO_uCl_v}\,(g) \quad \mathbf{(1)}$$

Then, the liquid-phase Na_xWO_y was volatilized to the gas phase, reacting with sulfur vapor to grow the monolayers WS_2 on the substrate:

$$\mathbf{Na_xWO_y}\,(l) + \mathbf{S}\,(g) \rightarrow \mathbf{WS_2}\,(s) + \mathbf{Na_2SO_3}\,(s) + \mathbf{SO_2}\,(g) \quad \mathbf{(2)}$$

In addition, sulfur vapor reacted with vapor WO_uCl_v to form WS_2:

$$\mathbf{WO_uCl_v}\,(g) + \mathbf{S}\,(g) \rightarrow \mathbf{WS_2}\,(s) + \mathbf{SO_2}\,(g) \quad \mathbf{(3)}$$

Similar to the case of MoO_3/NaCl mixture as a precursor for the MoS_2 growth,[2] the products of WO_3/NaCl mixture, both the molten Na_xWO_y and vapor WO_uCl_v, would contribute in the growth of WS_2 in CVD.

S3. COMSOL simulation

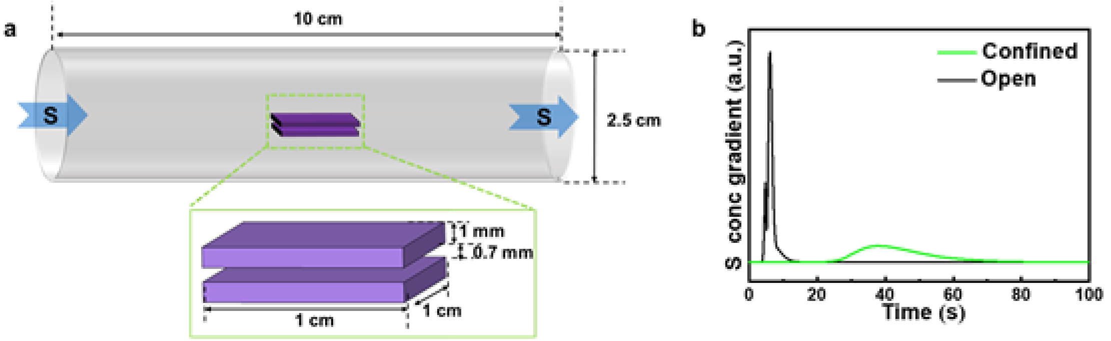

Figure S3. COMSOL model simulation. (a) The size of the geometric model for MSCS/confined mode. The model size for CCVD/open mode is similar except the aspectant substrate. (b) The evolution curve of S concentration gradient at the center of substrate for “confined” mode and “open” mode over time.

From **Figure S3b**, it is found that the concentration gradient of S in “confined” mode is much smaller than that in “open” mode, indicating a mild growth kinetics.

S4. Optimum mass ratio of $NaCl:WO_3$ for MSCS CVD

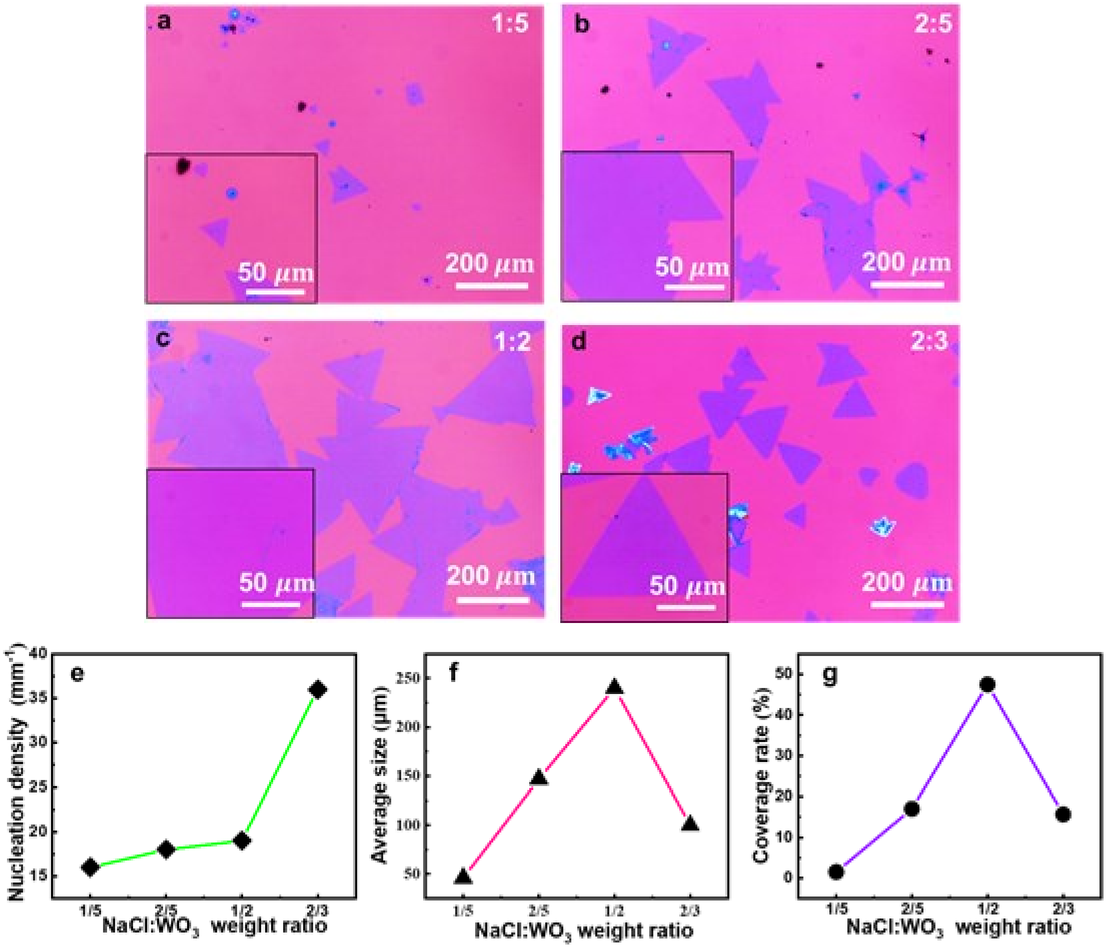

Figure S4. Impact of NaCl: WO_3 on the WS_2 growth. (a-d) Optical result of WS_2 sample with a gradually increased $NaCl:WO_3$ ratio (1:5, 2:5, 1:2 and 2:3). (e-g) nucleation density, average domain size and coverage rate of WS_2 as a function of WO_3 concentration.

As shown above, when the weight ratio of NaCl to WO_3 increases from 1:5 to 1:2, the average size of WS_2 sample increases from 50 μm to 250 μm, and the coverage rate increases from nearly 0 to ~50%; this indicates that more NaCl can significantly increase WS_2 sample size and coverage rate. However, with the increase of weight ratio to 2:3, the average size and coverage of WS_2 samples are reduced to ~100 μm and 15%, respectively; this indicates that excessive NaCl inhibits the growth of WS_2. Therefore, the inadequate NaCl ($NaCl:WO_3$ ratio ~1:5) could not react completely with the WO_3 precursors; this results in the small grain size of WS_2 with the unreacted WO_3 particles.

On the contrary, excessive NaCl (NaCl:WO_3 ratio $\geq$1:5) would lead to the fast evaporation of WO_3 source and unstable growth kinetics.[3]

S5. Growth experiments in open and confined modes

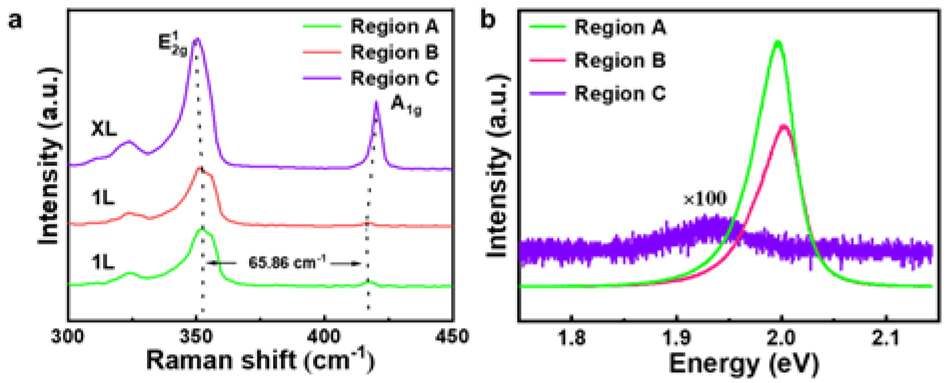

Figure S5. Spectroscopic characterizations of samples at Regions A-C. (a) Raman spectra and (b) Photoluminescence (PL) spectra of as-grown WS_2 flakes in **Figure 2c-I to III**. Dash lines trace the shifts of E_{2g}^{1} peak and A_{1g} peak of WS_2 monolayer and multilayer.

The Raman spectra in **Figure S5a/Figure 2d** are composed of two peaks at 350.71 cm^{-1} for E_{2g}^{1} and 416.57 cm^{-1} for A_{1g} in regions A and B. The difference between two modes is ~65.86 cm^{-1} which indicates the classic single layer WS_2 characteristics, as reported before.[4, 5] Different from the "covered" region, the Raman spectrum in Region C demonstrates that both peaks' intensity (A_{1g} and E_{2g}^{1}) and the ratio of A_{1g}/E_{2g}^{1} in region C (**Figure 2c-III**) are increased; these facts match well with the characters of multilayer WS_2.[6] Also, upon increasing the layer number, E_{2g}^{1} mode undergoes a red-shift while A_{1g} mode has a blue-shift with an enlarged width of the peak.[6] Apart from Raman spectra, the PL spectrum of **Figure S5b** in both A and B regions shows a strong peak at 1.97 eV (A excitation), as reported before; this observation confirms the direct band gap of the as-grown monolayer WS_2 (**Figure 2c-I and II**).[3, 6-8] Furthermore, the full width at half maxima (FWHM) of the PL peak region A is only 40 meV, which determines the high crystallinity of WS_2.[9] Compared to the confined regions (A and B), region C demonstrates the PL signal quenching due to the direct−indirect transition within multilayers in the bandgap of as-grown WS_2.[6] Therefore, regions A and B (MSCS system) are occupied with single-layered WS_2

plates while region C (CCVD system) is build up on multilayers, indicating quite different growth dynamics in their regions.

S6. Control experiments of CCVD

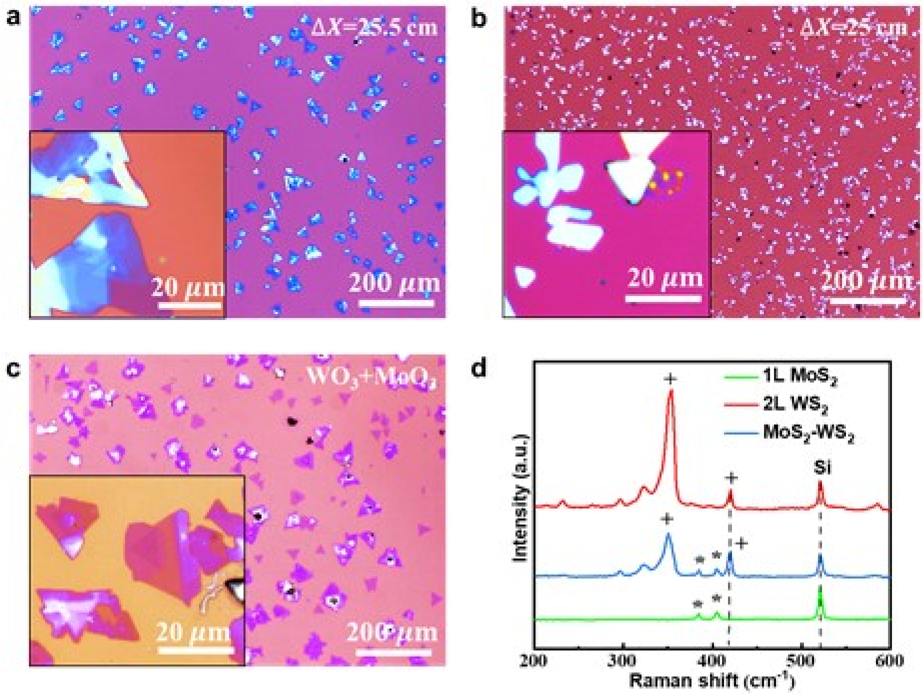

Figure S6. Control experiments of CCVD with the same experimental conditions for layer-number and heterostructure control growth of 2D WS_2 by MSCS. (a, b) Optical images of WS_2 sample prepared by CCVD method with different *ΔXs*. *ΔX* is the distance between the S source and substrate. (c) Optical images of MoS_2-WS_2 heterostructure sample based on CCVD method. The bottom insets of (a–c) show the magnified views of samples. (d) Raman spectra of multiple components in (c).

Different color contrasts in **Figure S6a** indicate different thicknesses from the flake, wherein the white flakes are multilayer (≥4 layer) WS_2 and the blue flakes relate to 1-3 layer(s). **Figure S6b** shows large number of white WS_2 plates which indicates its multilayer characteristic (≥4 layer). Therefore, the as-grown sample by CCVD always suffers from high S:W ratio with irregular multilayer flakes. Apart from WS_2 sample, according to **Figure S6c** and **d**, the as-grown sample by CCVD suffers from multiple components: 1L MoS_2, 2L WS_2, vertical and lateral MoS_2-WS_2 lateral heterostructures. In addition, the as-grown sample by CCVD shows much smaller domain size (<20 μm) than MSCS strategy (~50 μm), which in turn indicates that the MSCS method can promote the in-plane growth of MoS_2-WS_2 lateral heterostructures.

Noted that, the reaction parameters in Figure **S6a-c** are similar to MSCS method of 1L, 2L WS_2 and MoS_2-WS_2 lateral heterostructure.

S7. Transfer process of WS_2 sample for STEM test and STEM characterization of WS_2 sample

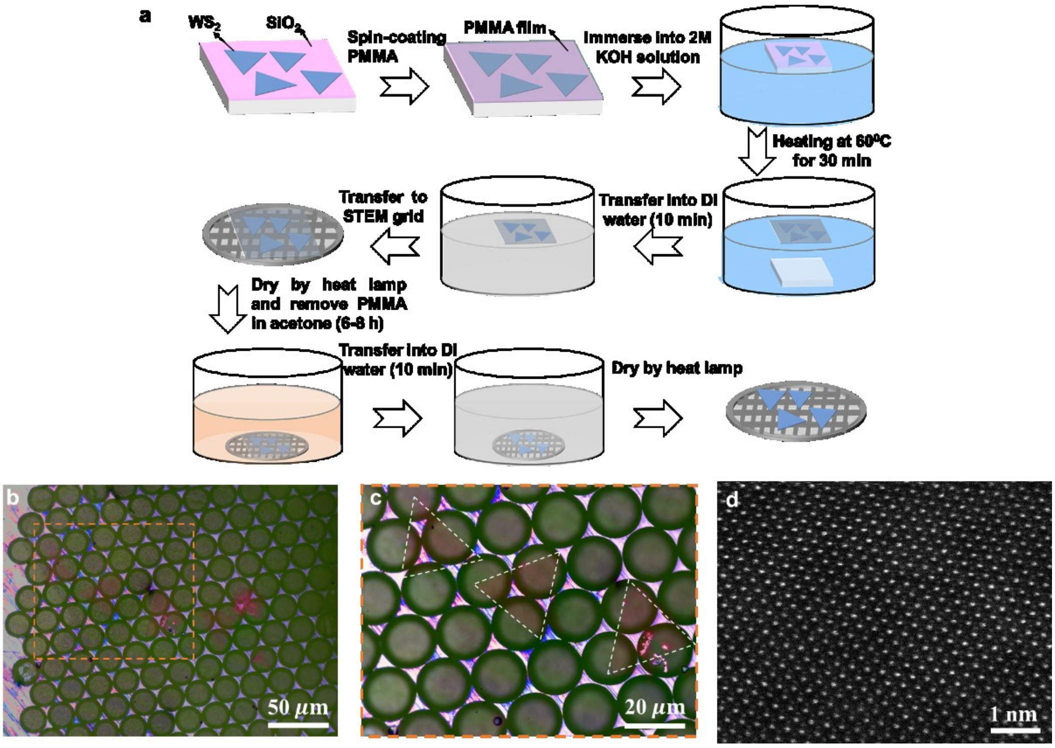

Figure S7. Preparation process of TEM sample and TEM observation results. (a) PMMA(polymethyl methacrylate) assist method of transfer WS_2 on a STEM grid. (b, c) Optical image of the WS_2 domain is transferred on a STEM grid, where (c) is the enlarged image of the yellow-dotted box in (b). (d) HAADF-STEM image of an as-grown WS_2 monolayer flake.

S8. Size modulation of 1L WS_2 plates

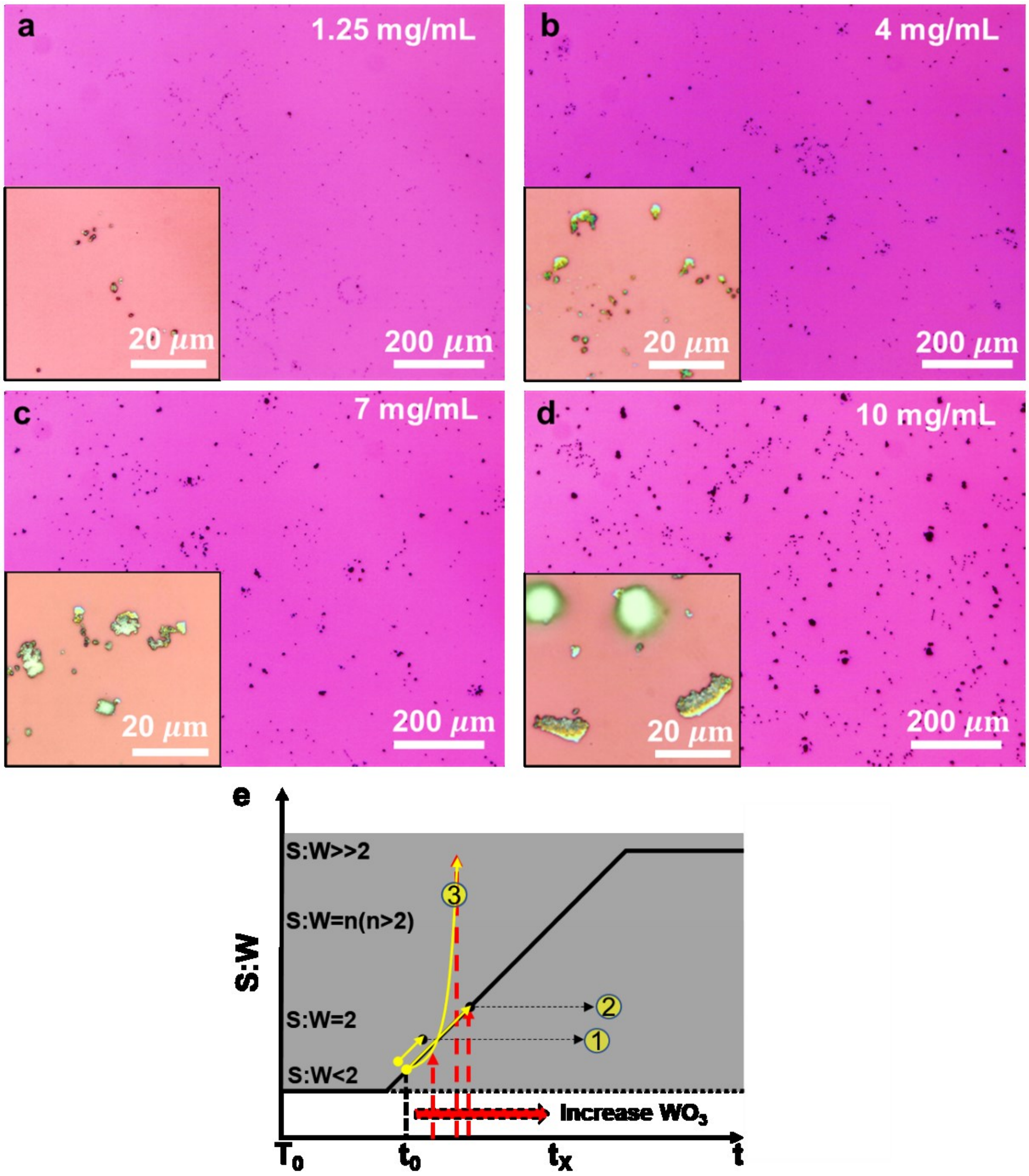

Figure S8. (a-d) Optical results of WO_3 particles with the WO_3 concentrations of 1.25, 4, 7 and 10 mg/mL. (e) GR evolution under increased concentration of WO_3.

It is obvious that the diameter of WO_3 particles increases gradually under elevating WO_3 concentration. Large WO_3 particles are hardly mixed with NaCl uniformly, resulting in an incomplete CVD reaction and WO_3 residuals.

It is noted that the high concentration WO_3 tends to aggregate to big size particles on the substrate (>10 μm, **Figure S8a-d**) and thus cannot be fully melted due to uneven

mixing with NaCl. Some WO_3 particles mix with more NaCl and thus result in initially high concentration of W vapor. They are consumed fast, however, some WO_3 particles mix with less NaCl and thus result in low concentration of W vapor. They are consumed in a slow rate, therefore, the corresponding GR3 in **Figure S8e** shows a curved profile. The WS_2 plates take the growth of GM-1L-S and GM-1L-F within a moderate time while GM-XL takes a long time. Together with the locally uneven supply of W vapor, the resulted chaotic growth of WS_2 plates has moderate sizes, irregular shapes, and partial multi-layers (dark blue parts in **Figure 3a-VI**). The growth at GM-XL results in high nucleation density and the larger the dwelling time, the higher the density of nucleation. Therefore, the products at the high concentration of WO_3 have a relatively high nucleation density.

S9 Growth results of 2D WS_2 plates at different gap values

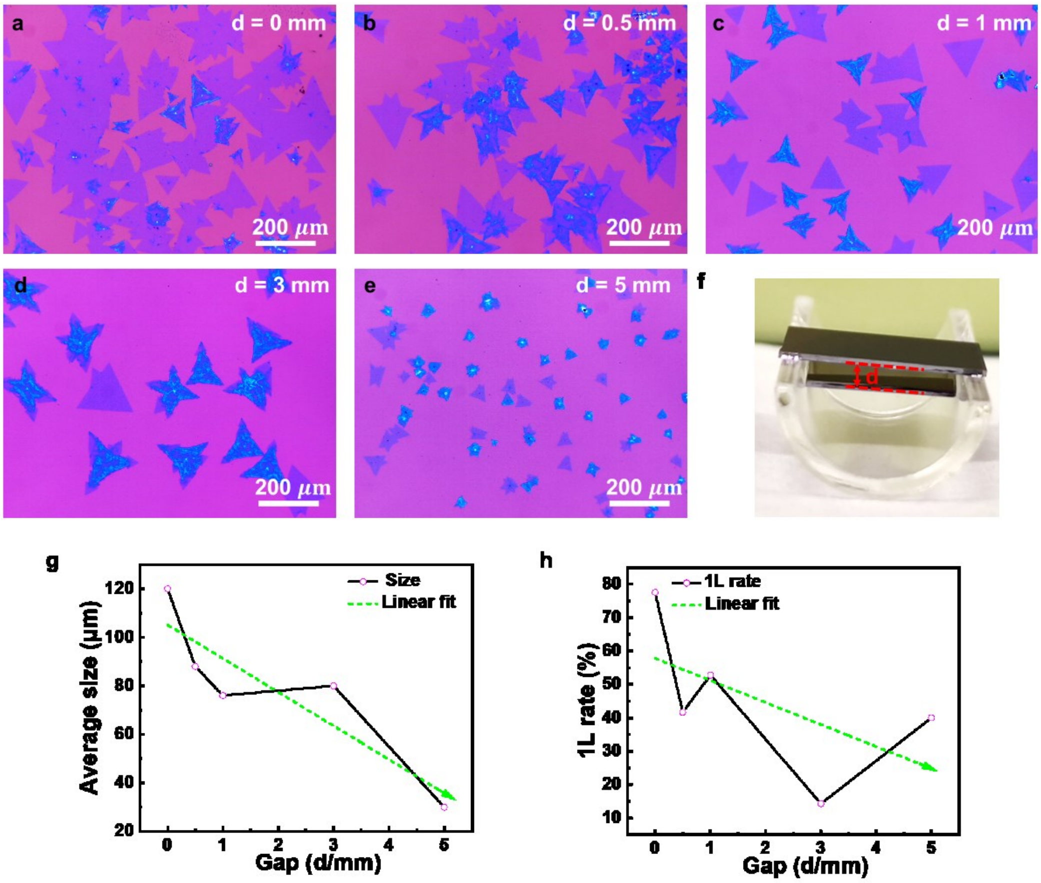

Figure S9. Growth results of 2D WS_2 plates at different gap values. (a-e) Optical images of WS_2 samples at different gaps between the two adjacent substrates. (f) The optical image of two adjacent substrates with a certain gap "d". (g) Statistical result of the average size as a function of gap value (a distance between the two adjacent substrates). (h) Statistical result of 1L rate (number of single layer flakes/total number of flakes) as a function of gap value.

According to **Figure S9**, the average size and 1L rate of WS_2 samples decrease with the increase of gap, as the number of non-uniform multilayer crystals increases. This proves that the S:W ratio increases with the increment of gap, which is consistent with the SW-T diagram in **Figure 3f**. The narrow gap favors the large size growth of WS_2 plates. Here, d = 0 mm does not mean that there is no gap between the two adjacent substrates, instead it refers to the separation by the mixture of WO_3 and NaCl with a rough distance of several micrometers.

S10. Growth of centimeter scale 1L WS_2 film by MSCS

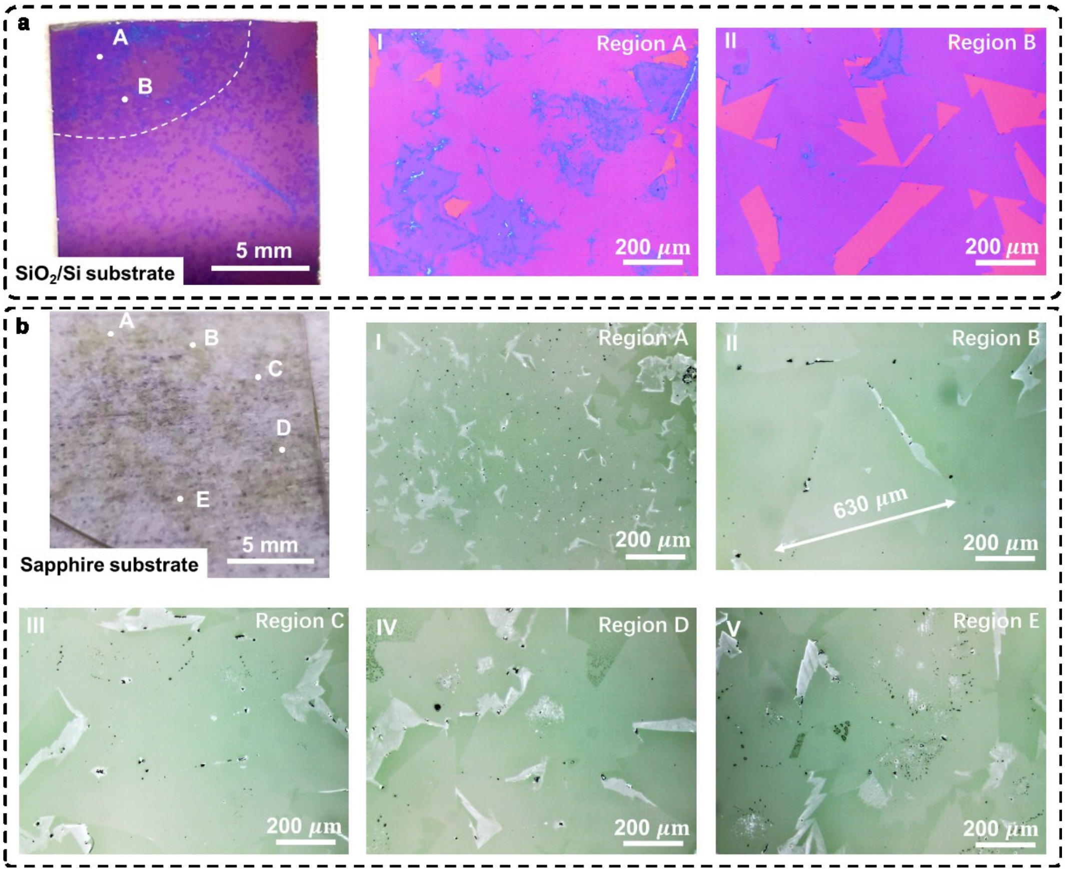

Figure S10. Large area growth of 1L WS_2 film on SiO_2/Si and sapphire substrates by MSCS CVD growth. (a) Optical images of the SiO_2/Si substrate with continuous WS_2 1L film and flakes. Two points (A and B) are specified for further observation and the corresponding magnified OM results are shown in (I) and (II). (b) Optical image of continuous WS_2 monolayer film with large flakes on ***c***-plane sapphire. Five points (A, B, C, D, and E) are specified for further observation and corresponding magnified OM results are shown in (I-V).

Single domains of 1L WS_2 on the SiO_2/Si substrate and *c*-plane sapphire demonstrate maximum sizes of ~472 and 630 μm, respectively, which are ranked at the top level among recent results (**Table S1**). Centimeter-scale 1L WS_2 continuous films are found on both SiO_2/Si and sapphire substrates.

S11. Large scale growth of 2L WS_2 film by MSCS

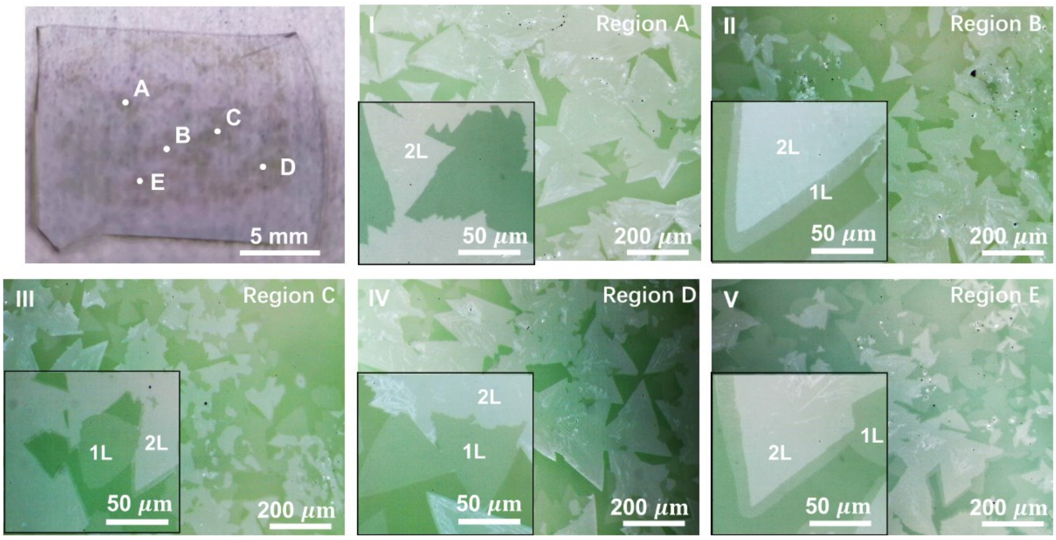

Figure S11. Large area growth of 2L WS_2 film/plates on sapphire substrates by MSCS CVD growth. Optical image of continuous WS_2 bilayer film with large flakes on the ***c***-plane sapphire after the MSCS CVD growth. Five points (A, B, C, D and E) are specified for observation and the corresponding magnified OM results are shown in (I-V).

The 2L WS_2 samples on ***c***-plane sapphire demonstrated good coverage and reproducibility. It was found that 1L and 2L WS_2 plates were mixed and 2L WS_2 plates merged into continuous films in some areas of the sapphire substrate.

S12. Controllable layer number growth of 2D WS_2 and MoS_2

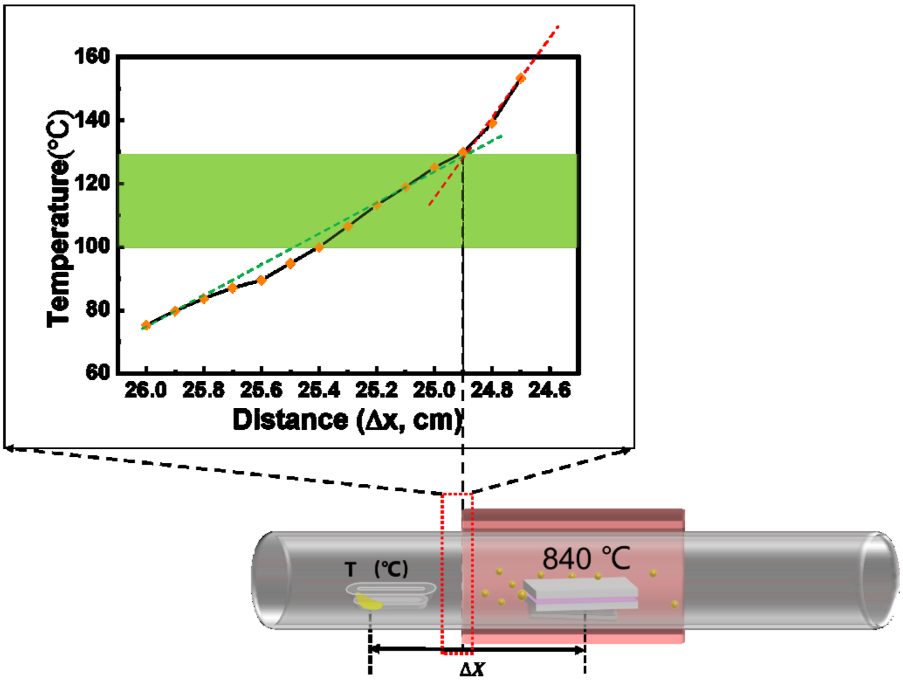

Figure S12. S source temperature vs. ΔX **when the central region of tubular furnace is heated up to 840 ℃.** The temperature is measured by a thermal couple under the same experimental conditions except there no S source is used in the quartz boat. Here, ΔX is the distance between S source and the substrate that is positioned at the center of the furnace. The black dotted line in the figure shows the boundary of quartz tube inside and outside of the furnace, where ΔX = 24.9 cm.

From **Figure S12**, it can be found that, with the decrement of ΔX, the temperature increases gradually, and the slope of temperature rise increases dramatically as the S source is positioned at the boundary (ΔX=24.9 cm).

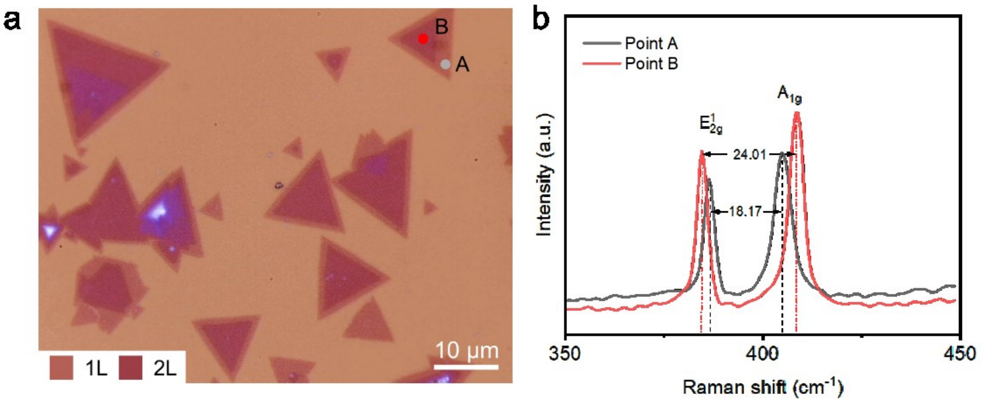

Figure S13. (a) Optical image and b) Raman spectra of selectively grown 2L MoS_2.

We successfully fabricated 2L MoS_2 selectively with similar strategy (MSCS) applied to WS_2, indicating the generality and validity of our proposed growth diagram for the growth of other 2D TMDCs. **Figure S13a** gives the optical images of several 2L MoS_2 plates, which are identified by Raman (**Figure S13b**). The sepia area of MoS_2 plates (point B) has a wide width of 24.1 cm^{-1} between E^1_{2g} and A_{1g} peaks, the 2L feature of MoS_2.[10]

S13. AFM and TEM characterizations of 2L WS_2

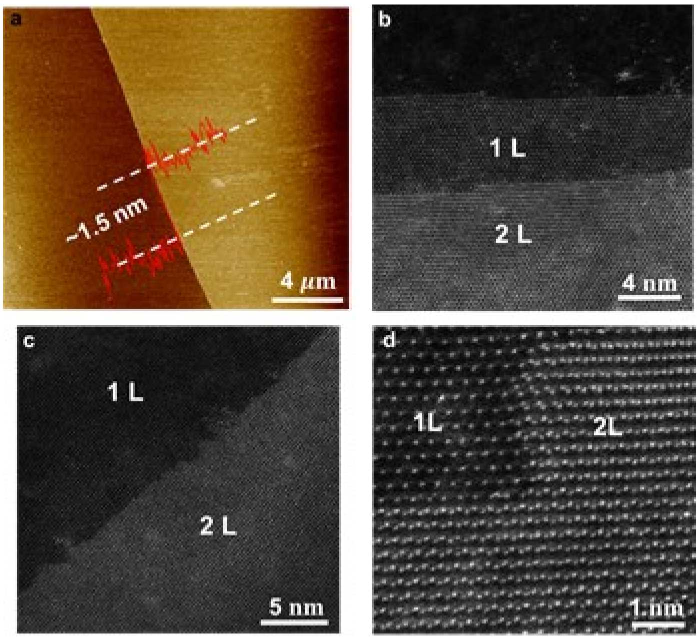

Figure S14. AFM and TEM characterizations of 2L WS_2. (a) The height topography of 2L WS_2 with no steps, which shows the thickness of the edge of WS_2 flake as ~1.5 nm. (b-d) HAADF-STEM image of an as-grown 1L-2L WS_2 flake.

Figure S14a reveals the thickness of WS_2 sample as ~1.5 nm, confirming the 2L feature of as-grown WS_2 film. **Figures S14b-d** show the HADDF-STEM image of the stepped area of a 1L−2L WS_2 plate; the 1L region displays a classic structure of 1L WS_2 as that in **Figure 2h**. The 2L region confirms a typical 3R phase structure of 2L WS_2, where extra W atoms reside on S atoms. Moreover, it can be found that the 1L/2L interface is not smooth, which suggests a fast and unstable dynamic growth of the second layer on the first layer.

S14. Optical characterization of MoS_2-WS_2 vertical heterostructure

The optical image, Raman of a single triangle plate (**Figure 5j and k**) displays clear contrast among 1L WS_2, 2L WS_2 and MoS_2-WS_2 vertical heterostructures, at 351cm^{-1} (E_{2g}^1 mode of WS_2, yellow), 416 cm^{-1} (A_{1g} mode of WS_2, blue), 384 cm^{-1} (E_{2g}^1 mode of MoS_2, ignitus) and 404 cm^{-1} (A_{1g} mode of WS_2, green). It is found that the E_{2g}^1 and A_{1g} modes of MoS_2 are distributed at the core of sample, while E_{2g}^1 and A_{1g} modes of WS_2 are clearly shown at the 2L WS_2 region (near point B). The 1L WS_2 region demonstrates weaker Raman intensity for both modes. In addition, the Raman point spectra from point C show a typical WS_2 monolayer characteristic (~65.3 cm^{-1} for the difference between E_{2g}^1 mode and A_{1g} mode), while the point B shows enhanced Raman intensity and enlarged wave number difference between E_{2g}^1 mode and A_{1g} mode (66 cm^{-1}). Moreover, point A shows the component of both WS_2 and MoS_2.

Apart from Raman mapping, the PL mapping from another MoS_2-WS_2 vertical junction sample in **Figure 5l** and **m** shows a clear contrast between 1L WS_2 and 2L WS_2/(MoS_2-WS_2 vertical junction). The 1L region shows a relatively strong PL emission at 630 nm (1.97 eV, red), while 2L region is nearly dark, indicating greatly suppressed PL due to the indirect band gap characteristics of 2L WS_2. However, we still obtain a weak signal of 1L MoS_2 from the core of sample (1.85 eV, cyan). In addition, the PL spectra in **Figure 5m** show the quenching of PL signals for both WS_2 and MoS_2, which further indicates the vertical heterostructure of MoS_2-WS_2 in region A.

S15 Comparison of as-grown 2D WS_2 plates by MSCS with previously reported results

Table S1. Reaction conditions and domain size of 1L WS_2

Carrier gas	Substrate	Precursor	Domain size (μm)	Ref.
Ar/H_2	Si/SiO_2	S and Na_2WO_4	3.68	[7]
Ar	Si/SiO_2	S and WO_3	1000	[11]
Ar/H_2	Si/SiO_2	$NaSO_4$ and WO_3	60	[12]
Ar/H_2	Si/SiO_2	S and WO_3	473	[6]
Ar	Au	H_2S and $H_4NO_{42}W_{12}$	420	[13]
Ar	Au	S and WO_3	500	[14]
Ar	Si/SiO_2	S and WO_3/NaCl	950	[15]
Ar	Si/SiO_2	S and WO_3/NaCl	472	**This work**
	Sapphire		630	

Table S2. Reaction conditions and domain size of 2L TMDCs

Product	Carrier gas	Substrate	Precursor	Average size (µm)	Ref.
MoS_2	Ar/H_2	NaF/SiO_2	MoO_3/NaF and H_2S	200	[16]
$MoSe_2$	Ar/H_2	Si/SiO_2	Se and WO_3	113	[17]
MoS_2	N_2/H_2	Si/SiO_2	S and MoO_3	300	[18]
WS_2			Se and WO_3	<50	
MoS_2	Ar	Soda line glass	S and MoO_3	200	[19]
WS_2	Ar	Si/SiO_2	H_2S and WO_3	~50	[20]
WS_2	Ar/H_2	Si/SiO_2	APCVD	**337**	**This work**

S16 Reaction parameters in MSCS CVD

Table S3. WO_3/NaCl suspension parameters with different mass ratios

**Weight ratio (WO_3: NaCl)**	**NaCl (mg)**	**WO_3 (mg)**	**DI Water (mL)**	**WO_3 concentration (mg/mL)**
**5:1**	4	20	4	5
**5:2**	8	20	4	5
**2:1**	10	20	4	5
**3:2**	~13.5	20	4	5

Table S4. WO_3 suspension parameters for size modulation

**WO_3 concentration (mg/mL)**	**NaCl (mg)**	**WO_3 (mg)**	**DI Water (mL)**
1.25	2.5	5	4
2.5	5	10	4
4	8	16	4
5	10	20	4
7	14	28	4
10	20	40	4

Table S5. WO_3/MoO_3 suspension parameters for side ratio modulation

**Weight ratio (WO_3: MoO_3)**	**NaCl (mg)**	**DI Water (mL)**	**WO_3 (mg)**	**MoO_3 (mg)**
20:3	10	4	20	3
10:1	10	4	20	2
20:1	10	4	20	1
50:1	10	4	20	0.4

References

[1] Y. Jin, Z. Zeng, Z. Xu, Y.-C. Lin, K. Bi, G. Shao, T.S. Hu, S. Wang, S. Li, K. Suenaga, H. Duan, Y. Feng, S. Liu, Chemistry of Materials 2019, 31 3534-3541.

[2] H.R. Rasouli, N. Mehmood, O. Cakiroglu, T.S. Kasirga, Nanoscale 2019, 11, 7317-7323.

[3] J. Wang, Y. Luo, X. Cai, R. Shi, W. Wang, T. Li, Z. Wu, X. Zhang, O. Peng, A. Amini, C. Tang, K. Liu, N. Wang, C. Cheng, Chemistry of Materials 2020, 32, 2508-2517.

[4] C. Cong, J. Shang, X. Wu, B. Cao, N. Peimyoo, C. Qiu, L. Sun, T. Yu, Advanced Optical Materials 2014, 2, 131-136.

[5] Y. Chen, L. Gan, H. Li, Y. Ma, T. Zhai, Adv Mater 2017, 29.

[6] A. Zafar, Z. Zafar, W. Zhao, J. Jiang, Y. Zhang, Y. Chen, J. Lu, Z. Ni, Advanced Functional Materials 2019.

[7] Y. Lu, T. Chen, G.H. Ryu, H. Huang, Y. Sheng, R.-J. Chang, J.H. Warner, ACS Applied Nano Materials 2018, 2, 369-378.

[8] J. Wang, M. Han, Q. Wang, Y. Ji, X. Zhang, R. Shi, Z. Wu, L. Zhang, A. Amini, L. Guo, N. Wang, J. Lin, C. Cheng, ACS Nano 2021, 15, 6633-6644.

[9] Y. Kobayashi, S. Sasaki, S. Mori, H. Hibino, Z. Liu, K. Watanabe, T. Taniguchi, K. Suenaga, Y. Maniwa, Y. Miyata, Acs Nano 2015, 9, 4056-4063.

[10] L. Li, I. Lee, D. Lim, M. Kang, G.-H. Kim, N. Aoki, Y. Ochiai, K. Watanabe, T. Taniguchi, Nanotechnology 2015, 26, 295702.

[11] F. Lan, R. Yang, S. Hao, B. Zhou, K. Sun, H. Cheng, S. Zhang, L. Li, L. Jin, Applied Surface Science 2020, 504.

[12] Y. Jin, M. Cheng, H. Liu, M. Ouzounian, T.S. Hu, B. You, G. Shao, X. Liu, Y. Liu, H. Li, S. Li, J. Guan, S. Liu, Chemistry of Materials 2020, 32, 5616-5625.

[13] S.J. Yun, Acs Nano 2015, 9, 5510-5519.

[14] Y. Gao, Z. Liu, D.M. Sun, L. Huang, L.P. Ma, L.C. Yin, T. Ma, Z. Zhang, X.L. Ma, L.M. Peng, H.M. Cheng, W. Ren, Nat Commun 2015, 6, 8569.

[15] X. Qiang, Y. Iwamoto, A. Watanabe, T. Kameyama, X. He, T. Kaneko, Y. Shibuta, T. Kato, Sci Rep 2021, 11, 22285.

[16] M.C. Chang, P.H. Ho, M.F. Tseng, F.Y. Lin, C.H. Hou, I.K. Lin, H. Wang, P.P. Huang, C.H. Chiang, Y.C. Yang, I.T. Wang, H.Y. Du, C.Y. Wen, J.J. Shyue, C.W. Chen, K.H. Chen, P.W. Chiu, L.C. Chen, Nat Commun 2020, 11, 3682.

[17] G. Peng, X. Yang, S. Wang, J. Zhang, G. Qi, S. Zhang, K. Liu, Z.H. Zhu, Z. Li, G. Wang, M. Zhu, S. Qin, ACS Appl Mater Interfaces 2020, 12, 23347-23355.

[18] X. Zhang, H. Nan, S. Xiao, X. Wan, X. Gu, A. Du, Z. Ni, K.K. Ostrikov, Nat Commun 2019, 10, 598.

[19] Q. Gao, Z. Zhang, X. Xu, J. Song, X. Li, Y. Wu, Nat Commun 2018, 9, 4778.

[20] B. Pan, K. Zhang, C. Ding, Z. Wu, Q. Fan, T. Luo, L. Zhang, C. Zou, S. Huang, ACS Appl Mater Interfaces 2020, 12, 35337-35344.
