# Supplementary figures and images for: Modified Spatially Confined Strategy Enabled Mild Growth Kinetics for Facile Growth Management of Atomically‐Thin Tungsten Disulfides
Source: Adv Sci (Weinh). 2022 Nov 29;10(3):2205638. doi: 10.1002/advs.202205638 (PMC9875684; doi:10.1002/advs.202205638)

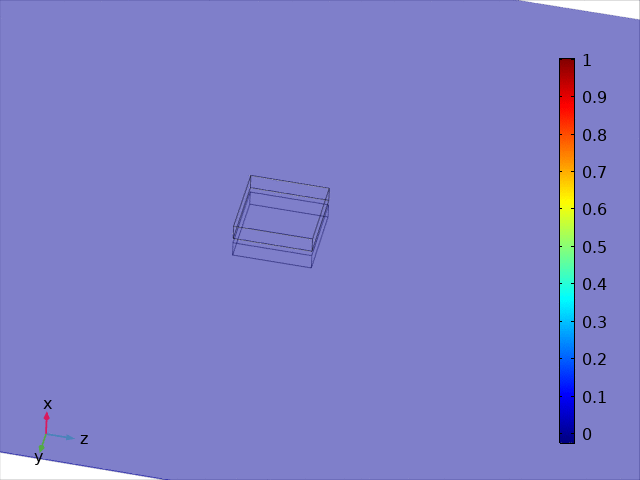

Supplement: Supplementary file 2 — Supporting Information [file ADVS-10-2205638-s001.gif]

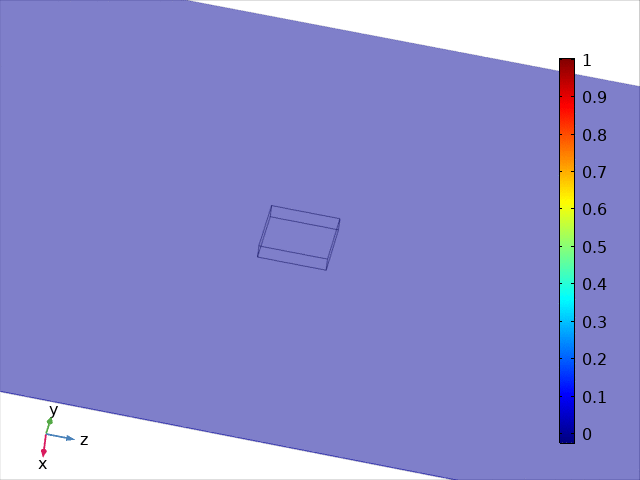

Supplement: Supplementary file 3 — Supporting Information [file ADVS-10-2205638-s004.gif]

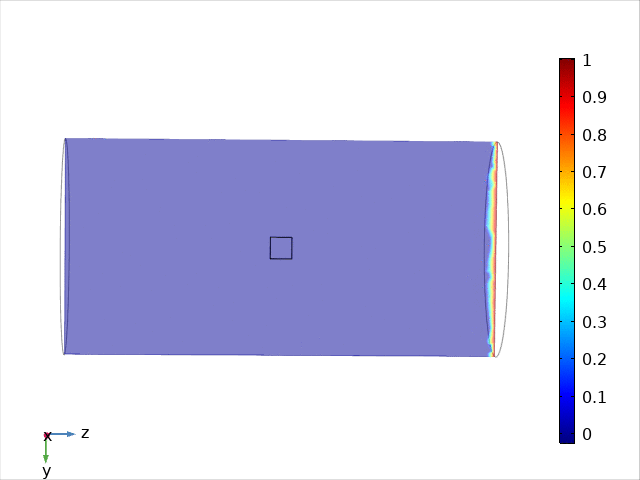

Supplement: Supplementary file 4 — Supporting Information [file ADVS-10-2205638-s003.gif]

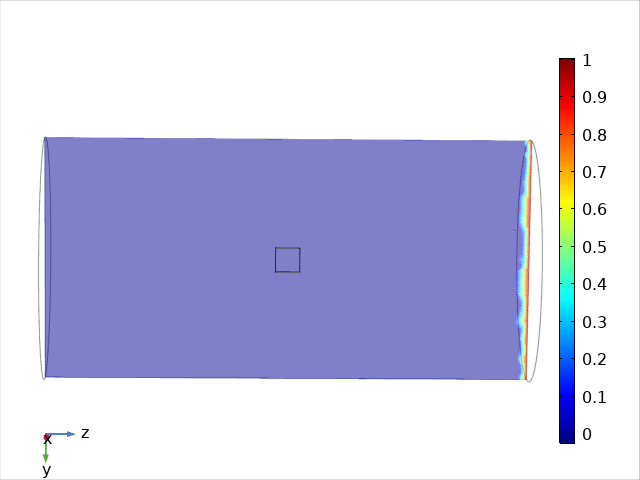

Supplement: Supplementary file 5 — Supporting Information [file ADVS-10-2205638-s002.gif]
